# Comparative evaluation of gene set analysis approaches for RNA-Seq data

**DOI:** 10.1186/s12859-014-0397-8

**Published:** 2014-12-05

**Authors:** Yasir Rahmatallah, Frank Emmert-Streib, Galina Glazko

**Affiliations:** Division of Biomedical Informatics, University of Arkansas for Medical Sciences, Little Rock, AR 72205 USA; Computational Biology and Machine Learning Laboratory, Center for Cancer Research and Cell Biology, School of Medicine, Dentistry and Biomedical Sciences, Queen’s University Belfast, 97 Lisburn Road, Belfast, BT9 7BL UK

## Abstract

**Background:**

Over the last few years transcriptome sequencing (RNA-Seq) has almost completely taken over microarrays for high-throughput studies of gene expression. Currently, the most popular use of RNA-Seq is to identify genes which are differentially expressed between two or more conditions. Despite the importance of Gene Set Analysis (GSA) in the interpretation of the results from RNA-Seq experiments, the limitations of GSA methods developed for microarrays in the context of RNA-Seq data are not well understood.

**Results:**

We provide a thorough evaluation of popular multivariate and gene-level self-contained GSA approaches on simulated and real RNA-Seq data. The multivariate approach employs multivariate non-parametric tests combined with popular normalizations for RNA-Seq data. The gene-level approach utilizes univariate tests designed for the analysis of RNA-Seq data to find gene-specific *P*-values and combines them into a pathway *P*-value using classical statistical techniques. Our results demonstrate that the Type I error rate and the power of multivariate tests depend only on the test statistics and are insensitive to the different normalizations. In general standard multivariate GSA tests detect pathways that do not have any bias in terms of pathways size, percentage of differentially expressed genes, or average gene length in a pathway. In contrast the Type I error rate and the power of gene-level GSA tests are heavily affected by the methods for combining *P*-values, and all aforementioned biases are present in detected pathways.

**Conclusions:**

Our result emphasizes the importance of using self-contained non-parametric multivariate tests for detecting differentially expressed pathways for RNA-Seq data and warns against applying gene-level GSA tests, especially because of their high level of Type I error rates for both, simulated and real data.

**Electronic supplementary material:**

The online version of this article (doi:10.1186/s12859-014-0397-8) contains supplementary material, which is available to authorized users.

## Background

Over the last few years transcriptome deep sequencing (RNA-Seq) has almost completely taken over microarrays for high-throughput studies of gene expression. In contrast to microarrays, RNA-Seq technology quantifies expression in counts of transcript reads mapped to a genomic region [1,2]. These read counts are integer numbers ranging from zero to millions. This is why approaches that were developed for the analysis of microarray data are generally not applicable to the analysis of RNA-Seq data: microarray approaches model the gene expression by continuous distributions. The most common use of RNA-Seq has been identifying genes that are differentially expressed (DE) between two or more conditions. Typically, gene counts are modeled using Poisson or Negative Binomial (NB) distribution, and several commonly used software packages such as edgeR [3], DESeq [4], and SamSeq [5] adapted for RNA-Seq, are freely available. Recently it was suggested to transform RNA-Seq count data prior to the analysis and apply normal-based microarray-like statistical methods, e.g. the limma pipeline [6] to RNA-Seq data [7].

Similarly, a decade ago, the focus of microarrays data analysis was also on finding DE genes. The methods for microarray data were dominated by univariate two-sample statistical tests for finding DE genes. However, it was quickly recognized that (1) biologically relevant genes with small changes in expression are almost always absent in the list of statistically significant DE genes, detected using two-sample tests with the correction for multiple testing [8], and (2) because genes do not work in isolation, statistical tests need to account for the multivariate nature of expression changes [9,10]. To address the shortcomings of gene-level analyses, conceptually new approaches were suggested which operated with gene sets, i.e. treating a gene set as an expression unit. Importantly, differentially expressed gene sets (such as biological pathways) incorporate existing biological knowledge into the analysis, thus providing more explanatory power than a long list of seemingly unrelated genes [9]. To date many methodologies for testing differential expression of gene sets have been suggested and are collectively named Gene Set Analysis (GSA) approaches [10-13].

GSA approaches can be either *competitive* or *self*-*contained*. Competitive approaches compare a gene set against its complement that contains all genes except the genes in the set, and self-contained approaches test whether a gene set is differentially expressed between two phenotypes [14,15]. Another technique that incorporates biological knowledge into the analysis, that requires a list of pre-selected DE genes to proceed, is the gene set over-representation analysis. Here, a set of pre-selected significantly DE genes is tested for over-representation in annotated gene sets such as Gene Ontology (GO) categories or Kyoto Encyclopedia of genes and genomes (KEGG) using standard statistical tests for enrichment [16]. A shortcoming of the over-representation approach is that it still requires a preselected gene list and genes with small changes may not be accounted for [10].

The first competitive GSA test for microarray data analysis (Gene Set Enrichment Analysis, GSEA [8]) was developed a decade ago, and in the last decade pathways analysis for microarray data has become a method of choice for explaining the biology underlying the experimental results [10,17,18]. One would expect there to be plenty of GSA approaches suitable for RNA-Seq data analysis, yet well-tested and justified methods are scarce. The first approach, adapting GSA for RNA-Seq data, was suggested by Young and colleagues [19]. They developed GOseq, a GO categories over-representation analysis that accounts for the over-detection of GO categories enriched with long and highly expressed genes in RNA-Seq data. Next, a non-parametric competitive GSA approach named GSVA (Gene Set Variation Analysis) has demonstrated highly correlated results between microarrays and RNA-Seq sets of samples of lymphoblastoids, cell lines which have been profiled by both technologies [20]. Shortly after, Wang and Cairns [21] suggested SeqGSEA, an adaptation of GSEA to RNA-Seq data. All of the aforementioned approaches are not without inherent biases: GO-Seq results depend on the methods selected for finding DE genes [19], and competitive approaches (in particular GSEA) are influenced by the filtering of the data and can even increase their power by the addition of unrelated data and noise [22].

The discussion about the possibility of using self-contained gene-level tests for GSA for microarrays data was on-going for a long time: such tests are straightforward and can be easily designed [11]. Some authors (e.g. [23,24]) were recommending to use gene-level tests for GSA. At the same time, because these tests are not truly multivariate and have much lower power compared to multivariate approaches, some authors [18] were advising against the application of gene-level tests for GSA. In a recent publication gene-level tests were claimed to be the first method of choice for GSA of RNA-Seq data [25]. In the simulation study expression data (reads) were taken from a multivariate normal distribution [25]. Because reads are integer numbers and are usually modeled using Poisson or Negative Binomial distribution, the simulation results of the study [25] may be inconclusive.

Thus far, except for gene-level GSA tests [25], the power and Type I error rates of self-contained approaches were not examined in the context of RNA-Seq data. Here we study the performance of several self-contained GSA approaches – multivariate and gene-level – for finding differentially expressed pathways in RNA-Seq data. The goals of our study are to: 1) describe several non-parametric multivariate GSA approaches developed for microarray data [18,26] that do not have distributional assumptions and are readily applicable to RNA-Seq data given proper normalization; 2) evaluate the performance of the four most commonly used RNA-Seq normalization approaches in combination with the aforementioned non-parametric multivariate GSA; 3) describe how univariate tests specifically designed for finding DE genes in RNA-Seq data can be extended to gene-level GSA tests by using procedures for combining genes *P*-values into a pathway *P*-value (Fisher’s combining probabilities Method (FM) [27], Stouffer’s Method (SM) [28] and the soft thresholding Gamma Method (GM) [25]); 4) evaluate the performance of the three most commonly used univariate tests for the analysis of RNA-Seq data (edgeR, DESeq, and eBayes) in combination with approaches for combining genes *P*-values into a pathway *P*-value; and 5) provide comparative power and Type I error rate analyses for multivariate and gene-level GSA tests.

In addition we evaluate whether non-parametric multivariate GSA approaches with different normalizations as well as gene-level GSA tests are prone to different types of selection biases. We check all GSA approaches for over-detection of pathways enriched with long genes. This bias was shown to exist in gene set over-representation analysis [19], but it is currently unknown whether it exists in GSA approaches. We also check whether GSA approaches over-detect pathways with small (large) number of genes and small (large) percentage of differentially expressed genes. In conclusion, we provide some recommendations for employing self-contained GSA approaches given RNA-Seq data.

In what follows we briefly describe several multivariate non-parametric tests [18,26]. We also consider the multivariate ROAST test [29] designed for microarray data but, given proper normalization, also applicable to RNA-Seq. Then we discuss approaches for combining *P*-values from univariate tests, such as edgeR, DESeq, and eBayes, specifically designed for the analysis of differential gene expression using RNA-Seq data sets into a pathway *P*-value. Approaches for RNA-Seq data normalizations together with a brief description of biological and simulated data used for testing purposes are presented in the end of this section.

## Methods

### Hypothesis testing

Statistically speaking the problem of finding differentially expressed pathways is a hypothesis testing problem. Consider two different phenotypes with *n*_1_ samples of measurements of *p* genes for the first and *n*_2_ samples of measurement of the same *p* genes for the second phenotype. Let the two *p*-dimensional random vectors of measurements ***X*** = (*X*_1_,…, *X*_*n*1)_ and ***Y*** = (*Y*_1_,…, *Y*_*n*2_) be independent and identically distributed with the distribution functions *F*, *G*, mean vectors *μ*_*x*_, *μ*_*y*_ and *p* × *p* covariance matrices *S*_*x*_ , *S*_*y*_. We consider the problem of testing the general hypothesis *H*_*0*_: *F* = *G* against an alternative *H*_1_: *F* ≠ *G*, or a restricted hypothesis *H*_*0*_: *μ*_*x*_ = *μ*_*y*_ against an alternative *H*_1_: *μ*_*x*_ ≠ *μ*_*y*_, depending on the test statistic.

### Multivariate tests

We adopted the multivariate generalization of the Wald-Wolfowitz (WW) and Kolmogorov-Smirnov (KS) tests [18] as suggested by Friedman and Rafsky [26]. These two tests were not used before in the context of pathway analysis with RNA-Seq data. The multivariate generalization is based on the minimum spanning tree (MST) of the complete network (graph) generated from gene expression data.

For an edge-weighted graph *G*(*V*,*E*) where *V* is the set of vertices and *E* is the set of edges, the MST is defined as the acyclic subset *T* ⊆ *E* that connects all vertices in *V* and whose total length ∑_*i*,*j* ∈ *T*_ *d*(*v*_*i*_, *v*_*j*_) is minimal. For the *p*-dimensional observations *X* and *Y*, an edge-weighted complete graph can be constructed with *N* nodes and *N*(*N*-1)/2 edge weights estimated by the Euclidean (or any other) distance measure between pairs of points in *R*^*p*^. The MST of such graph connects all *N* nodes (vertices) that are close in *R*^*p*^ with *N*-1 nodes.

For a univariate two-sample test (*p* = 1), the KS test begins by sorting the *N* = *n*_1_ + *n*_2_ observations in ascending order. Then, observations are ranked and the quantity *d*_*i*_ = *r*_*i*_/*n*_1_ − *s*_*i*_/*n*_2_ is calculated where *r*_*i*_ (*s*_*i*_) is the number of observations in *X* (*Y*) ranked lower than *i*, 1 ≤ *i* ≤ *N*. The test statistic is the maximal absolute difference *D* = *max*_*i*_|*d*_*i*_|, and *H*_*0*_: *μ*_*x*_ = *μ*_*y*_ is rejected for large *D*. The multivariate generalization of the KS test ranks multivariate observations based on their MST to obtain the strong relation between observations differences in ranks and their distances in *R*^*p*^. The MST is rooted at a node with the largest geodesic distance, and then the nodes are ranked in the high directed preorder (HDP) traversal of the tree [26]. Then, the test statistic *D* is found for the ranked nodes. The null distribution of *D* is estimated using samples label permutations, and *H*_*0*_: *μ*_*x*_ = *μ*_*y*_ is rejected for a large observed *D* [26].

For a univariate two-sample test (*p* = 1), the WW test begins by sorting the *N* = *n*_1_ + *n*_2_ observations in ascending order. Then, each observation is replaced by its phenotype label (*X* or *Y*), and the number of runs (*R*) is calculated where *R* is a consecutive sequence of identical labels. In the multivariate generalization of the WW test, all edges of MST incident between nodes belonging to different phenotype labels (*X* and *Y*) are removed, and the number of the remaining disjoint subtrees (*R*) is calculated. The permutation distribution of the standardized number of subtrees is asymptotically normal, and *H*_*0*_: *μ*_*x*_ = *μ*_*y*_ is rejected for a small number of subtrees [26].

We consider two other multivariate test statistics based on their high power and popularity. *N*-statistic [30,31] tests the most general hypothesis *H*_*0*_: *F* = *G* against a two-sided alternative *H*_1_: *F* ≠ *G*:

$$ {N}_{n_1{n}_2}=\frac{n_1{n}_2}{n_1+{n}_2}{\left[\frac{1}{n_1{n}_2}{\displaystyle \sum_{i=1}^{n_1}}{\displaystyle \sum_{j=1}^{n_2}}L\left({X}_i,{Y}_j\right)-\frac{1}{2{n}_1^2}{\displaystyle \sum_{i=1}^{n_1}}{\displaystyle \sum_{j=1}^{n_2}}L\left({X}_i,{X}_j\right)-\frac{1}{2{n}_2^2}{\displaystyle \sum_{i=1}^{n_1}}{\displaystyle \sum_{j=1}^{n_2}}L\left({Y}_i,{Y}_j\right)\right]}^{1/2} $$

Here we consider only *L*(*X*, *Y*) = ∥ *X* − *Y* ∥, the Euclidian distance in *R*^*p*^.

In the context of microarray data, a parametric multivariate rotation gene set test (ROAST) became popular for the self-contained GSA approaches [29]. ROAST uses the framework of linear models and tests whether, for all genes in a set, a particular contrast of the coefficients is non-zero [29]. It accounts for correlations between genes and has the flexibility of using different alternative hypotheses, testing whether the direction of changes for a gene in a set is *up*, *down* or *mixed* (up or down) [29]. For all comparisons implemented here the *mixed* hypothesis was selected. Applying ROAST to RNA-Seq data requires count normalization first. The VOOM normalization [7] was proposed specifically for this purpose where log counts, normalized for sequence depth, are used. In addition to counts normalization, VOOM calculates associated precision weights which can be incorporated into the linear modeling process within ROAST to eliminate the mean-variance trend in the normalized counts [7]. Considering that this feature is suited specifically for ROAST, we apply VOOM normalization with ROAST and do not apply any other normalization (except normalizing for gene length, see below).

### Combining *P*-values obtained using univariate tests for RNA-Seq

One way of designing a GSA test is to combine univariate statistics for individual genes [11,18]; we refer to this technique as ‘gene-level GSA’ in what follows. There are two popular univariate tests specifically designed for RNA-Seq data that rely on Negative Binomial model for read counts: edgeR [3] and DESeq [4]. Empirical Bayes method (eBayes [6]) correctly identifies hypervariable genes in the context of microarray data and, when adapted for RNA-Seq data through VOOM normalization [7], should be a powerful approach. Thus, in our comparative power analysis of gene-level GSA approaches, we include the following univariate tests: edgeR, DESeq, and eBayes. It should be noted that RNA-Seq counts are normalized for each test based on its recommended normalization method only.

The key question in designing a gene-level GSA test is how to combine statistics (*P*-values) from individual genes into a single gene set score (*P*-value). The problem of combining *P*-values has been recognized and studied for a long time (Fisher’s combining probabilities test [27]). Many methods for combining *P*-values are available and can usually be expressed in a form of *T* = ∑_*i*_*H*(*p*_*i*_), where *P*-vales are transformed by a function *H* [32]. In particular, Fisher’s method (FM) uses *H*(*p*_*i*_) = − 2*ln*(*p*_*i*_) and Stouffer’s method (SM) uses *H* to be the inverse normal distribution function [28].

Gamma Method (GM) is based on summing the transformed gene-level *P*-values using an inverse gamma cumulative distribution function ${G}_{w,1}^{-1}$ where *w* is the shape parameter, i.e. the combined test statistic is given by $T={\sum}_i{G}_{w,1}^{-1}\left(1-{p}_i\right)$ [33]. The shape parameter *w* controls the amount of emphasis given to gene-level *P*-values below a particular threshold. This feature is imposed by any transformation function *H* and is referred to as soft truncation threshold (STT) [33]. It is useful when there is pronounced heterogeneity in effects. The STT is controlled by *w* such that $w={G}_{w,1}^{-1}\left(1-STT\right)$. When *w* is large, GM becomes equivalent to the inverse normal Stouffer’s method which has *STT* = 0.5, and when it is 1 it becomes equivalent to Fisher’s method with *STT* = 1/*e*. Fridley *et al*. examined the performance of GM with various STT values and reported that STT values between 0.01 and 0.36 tend to give the best power [25]. For our study we chose *w* = 0.0137 that gives *STT* = 0.5. (For more detailed description of the methods for combining *P*-values see Additional file 1).

### Approaches to normalize RNA-Seq data before applying multivariate tests

Similar to microarray data [34,35], RNA-Seq data should be properly normalized before any further statistical tests can be applied. Raw counts are neither directly comparable between genes within one sample, nor between samples for the same gene. The counts of each gene are expected to be proportional to both gene abundance and gene length because longer genes produce more reads in the sequencing process. The counts will also vary between samples as a result of differences in the total number of mapped counts per sample (library size or sequencing depth). The first normalization for RNA-Seq data, ‘reads per kilobase per million’ (RPKM), was suggested by Mortazavi *et al*. [36] and was supposed to guard against over-detection of longer and more highly expressed genes. Recently, it was found that RPKM tends to identify weakly expressed genes as differentially expressed [37] and is not able to remove the length bias properly [19,37]. Oshlack and Wakefield [38] have demonstrated that the *t*-test power has a dependency on the square root of gene length even after RPKM normalization. While RPKM remains very popular, a number of other normalizations were suggested [4,39-41]. We employed three frequently used RNA-Seq normalization strategies to examine the performance of multivariate tests: the read per kilobase per million (RPKM) [36], the quantile-quantile normalization (QQN) [40], and the trimmed mean of M-values (TMM) [39]. Since both QQN and TMM ignore gene lengths, they are followed by RPKM to account for within-sample differences (see Additional file 1).

Instead of searching for better normalization, an alternative way of analyzing RNA-Seq data is to find a count data transformation such that all approaches developed for microarray data will become applicable [7]. It was shown that log counts, normalized for sequence depth, serve perfectly for this purpose when finding DE genes (VOOM [7] function in the limma package [6]). Since VOOM achieves between-samples normalization only, we followed it with RPKM normalization to account for gene length differences. VOOM returns normalized data in a log scale, so, before applying the RPKM normalization, the data were back-transformed to a linear scale.

Importantly, none of these normalizations (except RPKM for GO analysis [19]) have been tested in the context of GSA approaches. Here we provide the comparative power analysis of multivariate GSA approaches relying on the four aforementioned normalizations.

### Sample permutation

The null distribution of the test statistics used for the WW, KS, and *N*-statistic tests are estimated using sample permutations where sample phenotype labels (*X* and *Y*) are permuted randomly and the test statistic is calculated many times. To get reasonable estimates here this process was repeated 1,000 times. The empirical estimate of a *P*-value for a gene set is then taken as the proportion of permutations yielding a test statistic more extreme than the observed one from the original gene set. The same procedure was employed to compute the combined *P*-value *P*_*c*_ for a gene set after gene-level *P*-values are transformed and combined. This is necessary due to the lack of independence between genes which renders the parametric approach inaccurate.

### Biological data and pathways

We analyzed the subset of the data from Pickrell *et al*. [42], the sequenced RNA from lymphoblastoid cell lines (LCL) in 69 Nigerian individuals. We selected 58 unrelated individuals (parents), 29 males and 29 females. Pickrell *et al*. [42] dataset (the ‘Nigerian dataset’ in what follows) is attractive because there are two natural sets of True Positives: genes that are escaping X-chromosome inactivation and are therefore overexpressed in females (XiE), and genes that are located on male-specific region of Y chromosome and are therefore overexpressed in males (msY). The dataset also contains a natural set of False Positives: all X-linked genes that are not escaping inactivation (Xi, 387 genes after filtering). See Additional file 1 for more details.

Gene counts were obtained by detecting the overlaps between mapped short reads and the list of genomic ranges (of exons) under each gene using the Bioconductor GenomicRanges package (version 1.12.5). Short reads, which have non-unique mappings, were discarded. After filtering, the resulting count matrix had a total of 13,191 annotated genes and 58 samples. The normalized counts were transformed to log-scale using the function log_2_(1 + *Y*_*ij*_) to further reduce the effects of outliers. (For more detailed description of the Pickrell *et al*. [42] data preprocessing steps see Additional file 1).

Except Xi, msY, and XiE other gene sets were taken from the C2 pathways set of the molecular signature database (MSigDB) [43]. These gene sets were curated from online databases, biomedical literature, and knowledge of domain experts. Genes not present in the filtered dataset were discarded, and only pathways with the number of genes (*p*) in the range of 10 ≤ *p* ≤ 500 were included. The resulted dataset comprised 12,051 genes and 4,020 pathways. One C2 pathway, DISTECHE_ESCAPED_FROM_X_INACTIVATION (DEX), contains 13 X-linked genes found in our filtered dataset that were reported to escape inactivation [44]. While we can’t be sure if the other C2 pathways are differentially expressed between males and females, we expect that at least the three aforementioned pathways (msY, XiE and DEX) should be, and the Xi pathway should not be detected by any GSA test. Additional file 2 provides lists of all the genes and their descriptions in msY, XiE, DEX and Xi gene sets.

### Simulation of RNA-Seq counts

We model the count for a gene *i* in sample *j* by a random variable *Y*_*ij*_ from Negative Binomial (NB) distribution *Y*_*ij*_ ~ *NB*(*mean* = *μ*_*ij*_, *var* = *μ*_*ij*_(1 + *μ*_*ij*_*φ*_*ij*_)) = *NB*(*μ*_*ij*_, *φ*_*ij*_), where *μ*_*ij*_ and *φ*_*ij*_ are respectively the mean count and dispersion parameter of gene *i* in sample *j*. For each gene in a gene set, a vector of mean counts, dispersion, and gene length information (*μ*_*i*_, *φ*_*i*_, *L*_*i*_), is randomly selected from a pool of vectors derived from the processed Nigerian dataset (see Additional file 1). The dispersion parameter for each gene was estimated using the Bioconductor package edgeR (version 3.4.2) by the empirical Bayes method [45]. Counts, normalized using different approaches, were transformed to log-scale using the transformation function log_2_(1 + *Y*_*ij*_) to further reduce the effects of outliers. Additional file 3: Figure S2 and S3 show the density and histogram plots for the original counts and NB simulated counts before and after different normalizations. The simulated counts match the original counts reasonably well.

To evaluate the tests performance as accurately as possible, simulation experiments should mimic real expression data as closely as possible. In a real biological setting, not all genes in a gene set are differentially expressed, and the fold changes of genes between different phenotypes can vary. Therefore, we introduced two parameters: *γ*, the percentage of genes truly differentially expressed in a gene set; and *FC*, the amount of fold change in gene counts between two phenotypes. These parameters are expected to influence the power of different tests on a different scale. For the *γ* parameter, we consider *γ*∈{1/8, 1/4, 1/2}, and for the parameter *FC*, the values span the range from 1.2 to 3. Using simulations we assess the detection power for all tests by testing the hypothesis *H*_*0*_: *μ*_*x*_ = *μ*_*y*_ (or *H*_*0*_: *FC* = 1) against an alternative *H*_1_: *μ*_*x*_ ≠ *μ*_*y*_ (or *H*_1_: *FC* ≠ 1).

We simulated two datasets of equal sample size, *N*/2 (*N* = 20 and *N* = 40) forming 1,000 non-overlapping gene sets, each constructed from *p* random realizations of NB distribution. These two datasets represent two biological conditions with different outcomes. For a gene set in one phenotype, we generate *p* random realizations of NB distribution with parameters (*μ*_*i*_, *φ*_*i*_). For the same gene set in the second phenotype, we generate NB realizations with parameters (*FC μ*_*i*_, *φ*_*i*_) when *i* ≤ *γp* represents DE genes and NB realizations with parameters (*μ*_*i*_, *φ*_*i*_) when *i* > *γp* represents non-DE genes. Two cases were considered in our simulations: when the number of genes in a gene set is relatively small (*p* = 16) or when the number is relatively large (*p* = 100). To avoid having all the DE genes up-regulated for all generated gene sets in one phenotype, we swapped the generated counts for half of the DE genes (1 ≤ *i* ≤ *γp*/2) between the two phenotypes. Hence, now in each generated gene set, half of the DE genes are up-regulated and half are down-regulated between the two phenotypes. This will also avoid the problem of having large differences in total counts per sample between the two phenotypes.

To estimate the Type I error rates for all tests using simulated count data, we set *FC* and *γ* to 1 and simulated two datasets of equal sample size, *N*/2 (*N*∈{20,40,60}) from 1,000 gene sets, each constructed form *p* random realizations of Negative Binomial distribution with parameters (*μ*_*i*_, *φ*_*i*_) where *p*∈{16,60,100}. Then, we estimate the proportion of gene sets that reject *H*_*0*_: *μ*_*x*_ = *μ*_*y*_ (or *H*_*0*_: *FC* = 1) among the 1,000 generated sets.

## Results

### Simulation study

#### Type I error rate

Table 1 presents the estimates of the attained significant levels for the multivariate tests with different normalizations. As expected as the sample size *N* increases, the Type I error rates decrease. When the sample size is small (*N* = 20), *N*-statistic with VOOM normalization gives the most conservative Type I error rate, followed by ROAST (for *p* = 16, 60). This can be explained by VOOM’s ability to model the mean-variance relationship of count data for small *N*. But when the sample size is larger, TMM almost always gives more conservative estimates than VOOM (except when *N* = 60, *p* = 100). WW seems to be the most liberal among multivariate tests, followed by KS. For every test the Type I error rate is virtually unaffected when the number of genes in a pathway (*p*) increases.

**Table 1:** **Type I error rates for multivariate methods, α = 0.05**

		***p*** **=** **16**				***p*** **=** **60**				***p*** **=** **100**			
		**RPKM**	**QQN**	**TMM**	**VOOM**	**RPKM**	**QQN**	**TMM**	**VOOM**	**RPKM**	**QQN**	**TMM**	**VOOM**
*N* = 20	N-stat	0.060	0.062	0.038	0.038	0.054	0.062	0.058	0.035	0.052	0.050	0.049	0.047
	WW	0.096	0.103	0.091	0.091	0.102	0.102	0.096	0.070	0.097	0.099	0.099	0.089
	KS	0.104	0.090	0.083	0.082	0.102	0.088	0.077	0.076	0.080	0.077	0.072	0.092
	ROAST				0.050				0.048				0.036
*N* = 40	N	0.053	0.048	0.049	0.048	0.058	0.052	0.035	0.048	0.054	0.047	0.039	0.051
	WW	0.066	0.075	0.063	0.073	0.060	0.058	0.056	0.076	0.056	0.067	0.067	0.079
	KS	0.069	0.071	0.072	0.073	0.068	0.079	0.059	0.059	0.055	0.066	0.081	0.065
	ROAST				0.052				0.050				0.039
*N* = 60	N	0.052	0.054	0.060	0.067	0.051	0.040	0.053	0.055	0.046	0.054	0.059	0.044
	WW	0.089	0.066	0.065	0.079	0.057	0.069	0.060	0.073	0.065	0.065	0.076	0.064
	KS	0.061	0.073	0.055	0.060	0.052	0.059	0.061	0.070	0.053	0.051	0.068	0.047
	ROAST				0.054				0.043				0.055

**Figure 1:**
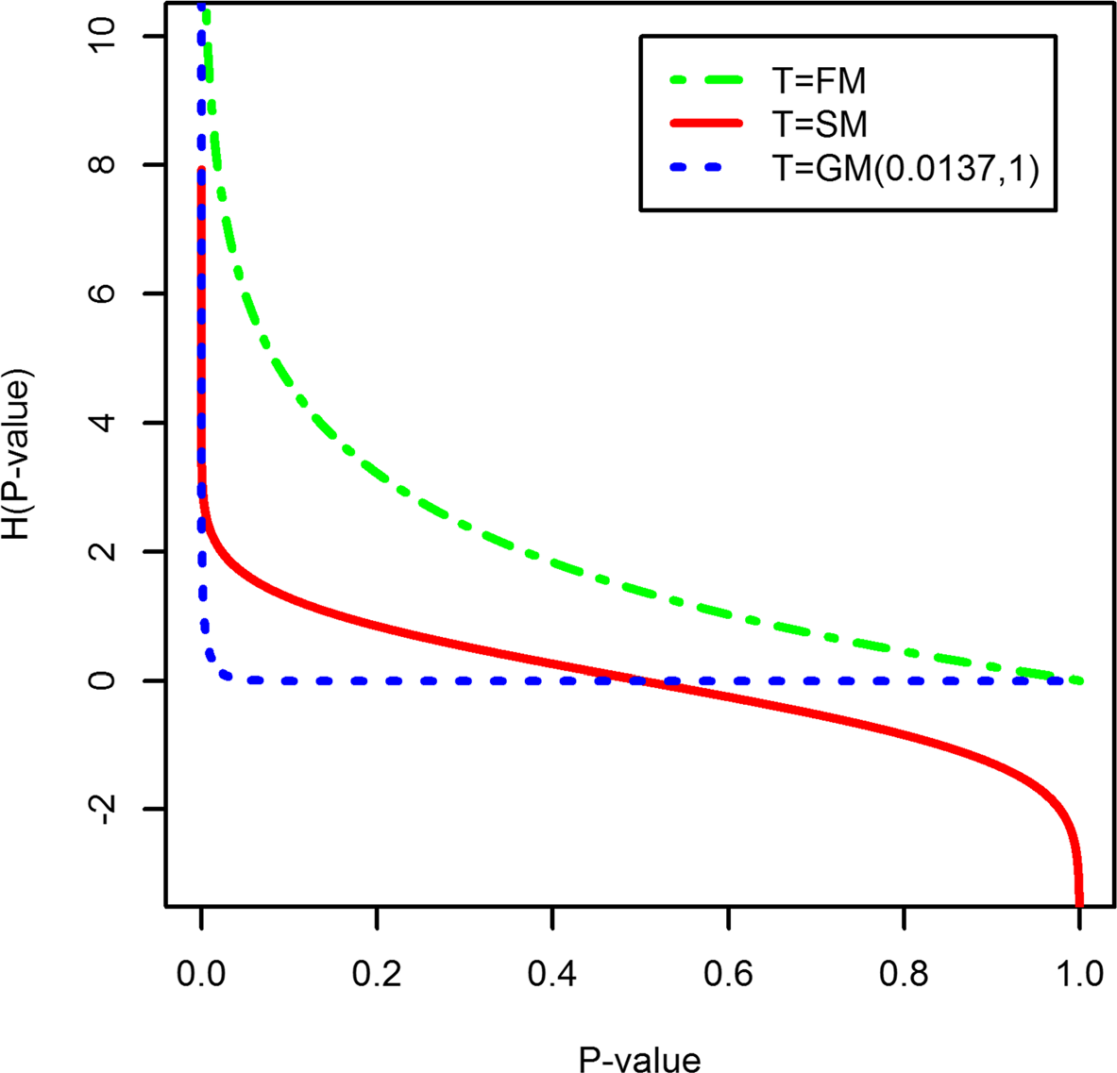
**The functional relationship between the transformed and the original** ***P-*** **values for different transformation functions** ***H*** **(used by FM,** **SM and GM with STT** **=** **0.05).**

We next consider Type I error rates for gene-level GSA tests that use univariate RNA-Seq specific tests (edgeR, DESeq and eBayes) and employ different methods for combining *P*-values (FM, SM and GM with STT = 0.05). To better understand the functional relationship between the transformed and the original *P*-values we applied the transformation functions *H* (used by FM, SM and GM with STT = 0.05) to a range of *P*-values (*P*-value is changing from 10^−5^ to 1 with the step of 10^−5^, Figure 1).

Figure 1 shows interesting biases that are introduced by different transformations (FM, SM and GM). First, GM is only sensitive to the extremely small *P*-values and virtually ignores all the others. In practice it means that gene sets with a large number of genes will be called DE by tests with GM more frequently than gene sets with a small number of genes. This is expected because, by pure chance alone, gene sets with a large number of genes have higher probability to contain genes with extremely small *P*-values, and GM ignores all the others. Second, FM accounts not only for the extremely small *P*-values, but also for generally small *P*-values, as well as large *P*-values. Therefore, tests with FM would call a gene set DE if and only if most of the genes in a gene set have small *P*-values. Gene sets with a large number of genes will be called DE by tests with FM less frequently than gene sets with a small number of genes, because, again, by pure chance alone, gene sets with a large number of genes have higher probability to contain genes with large *P*-values and large *P*-values affect the FM score (Figure 1). Third, unlike FM and GM, SM maps *P*-values less than 0.5 and greater than 0.5 to positive and negative values with magnitudes depending on the deviation from 0.5 (Figure 1). As a result tests with SM would call a gene set DE if and only if all genes in a set have small *P*-values. Similar to tests with FM, tests with SM are expected to call DE gene sets with a small number of genes.

The simulation results clearly demonstrate that the Type I error rates are influenced by the aforementioned biases introduced by different transformation functions (FM, SM and GM). As expected, for all gene-level GSA approaches that use univariate tests and different transformation functions to combine *P*-values, tests with GM show the highest Type I error, followed by tests with FM and SM (Table 2, Figure 1). Also, for any *P*-values combining method, edgeR shows the highest Type I error, followed by DESeq and eBayes respectively. In addition, with GM transformation, when the number of genes in a gene set (*p*) increases, especially for edgeR and DESeq, the Type I error rate becomes extremely high.

**Table 2:** **Type I error rates for gene-level GSA methods, α = 0.05**

		***p*** **=** **16**			***p*** **=** **60**			***p*** **=** **100**		
		**edgeR**	**DESeq**	**eBayes**	**edgeR**	**DESeq**	**eBayes**	**edgeR**	**DESeq**	**eBayes**
*N* = 20	FM	0.087	0.067	0.045	0.107	0.072	0.046	0.096	0.065	0.037
	SM	0.052	0.048	0.045	0.063	0.062	0.048	0.046	0.047	0.040
	GM	0.123	0.092	0.049	0.187	0.141	0.039	0.245	0.180	0.041
*N* = 40	FM	0.067	0.058	0.049	0.082	0.059	0.049	0.090	0.073	0.054
	SM	0.065	0.062	0.054	0.059	0.060	0.053	0.063	0.061	0.057
	GM	0.092	0.063	0.051	0.132	0.091	0.058	0.164	0.104	0.051
*N* = 60	FM	0.066	0.061	0.048	0.056	0.050	0.049	0.072	0.061	0.044
	SM	0.052	0.047	0.046	0.048	0.050	0.049	0.048	0.046	0.058
	GM	0.088	0.072	0.049	0.090	0.065	0.050	0.108	0.091	0.046

#### The power to detect shift alternatives

**Figure 2:**
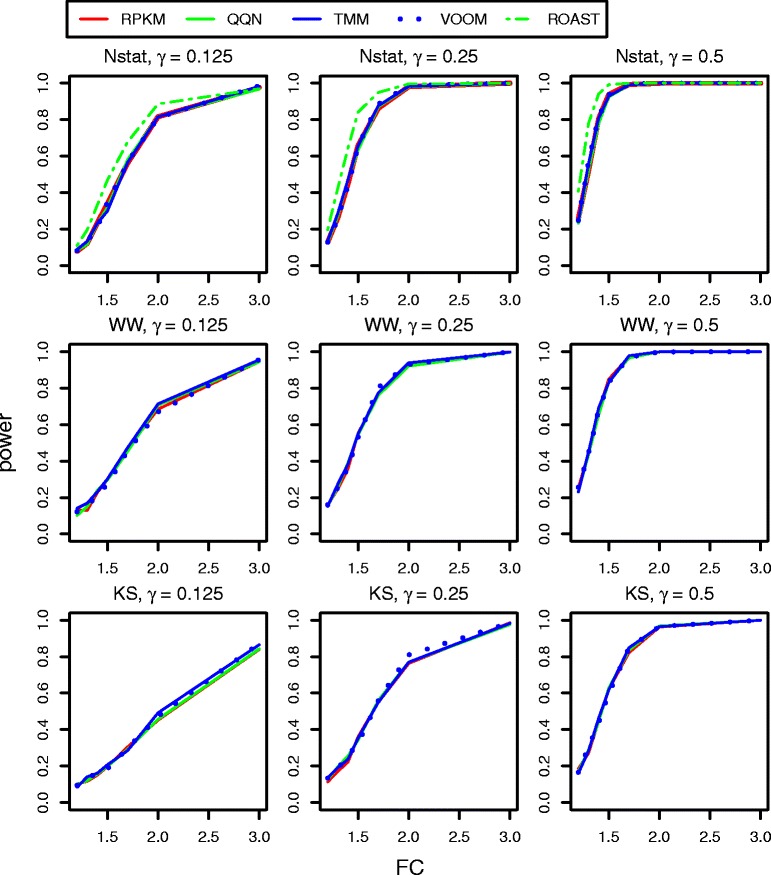
**The power curves of multivariate tests with different normalizations when shift alternative hypothesis** **(** ***H***_**1**_ **)** **holds true and the number of genes in pathways** ***p*** **=** **16** ***(N*** **=** **20).**

Figure 2 presents the power estimates for the *N*-statistic, WW and KS multivariate tests with different normalizations and ROAST with only VOOM followed by RPKM normalization (see Section Multivariate tests), when *H*_1_: *μ*_*x*_ ≠ *μ*_*y*_ is true (*N* = 20, *p* = 16). It appears that ROAST outperforms all the other approaches followed respectively by the *N*-statistic, WW, and KS. Different normalizations do not affect the tests’ power at all (Figure 2). When *N* = 20 and *p* = 100 (Additional file 3: Figure S3), *N* = 40 and *p* = 16 (Additional file 3: Figure S4), *N* = 40 and *p* = 100 (Additional file 3: Figure S5) the results are similar, but the power to detect even small fold changes is higher for all tests.

**Figure 3:**
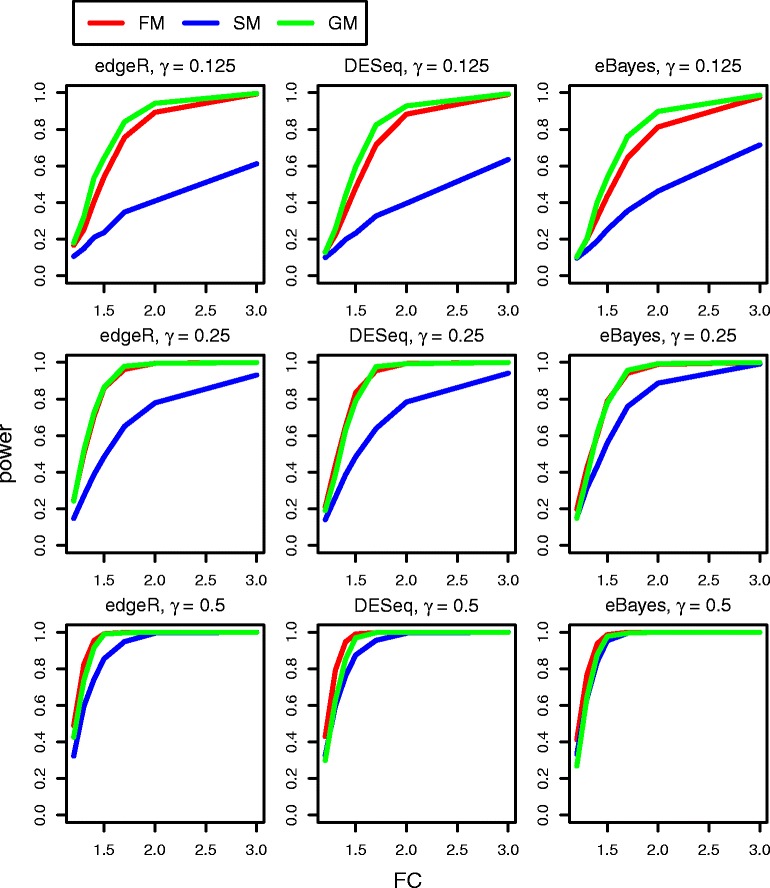
**The power curves of gene** **-** **level GSA methods when shift alternative hypothesis** **(** ***H***_**1**_ **)** **holds true and the number of genes in pathways** ***p*** **=** **16** **(** ***N*** **=** **20).**

Figure 3 presents the power estimates for gene-level GSA approaches that use univariate tests (edgeR, DESeq, and eBayes) and employ different methods for combining *P*-values (FM, SM, and GM with STT = 0.05) when *H*_1_ is true (*N* = 20, *p* = 16). When the percentage of truly differentially expressed genes is small (*γ* = 1/8), all three tests that apply GM have slightly higher power than those tests with FM, while the power of tests with SM is much smaller. When *γ* increases (from the top to the bottom on each panel of Figure 3) the difference between tests with GM and tests with FM diminishes, and the power of tests with SM becomes very close to the power of tests with FM and GM. The results when *N* = 20 and *p* = 100 (Additional file 3: Figure S6), *N* = 40 and *p* = 16 (Additional file 3: Figure S7) and *N* = 40 and *p* = 100 (Additional file 3: Figure S8) are similar, but the power to detect even small fold changes is higher for all tests. Comparing the performance of the three univariate tests under each *P*-value combining method shows that edgeR has slightly higher power than DESeq and eBayes, with both FM and GM, while eBayes has slightly higher power than edgeR and DESeq with SM (Additional file 3: Figure S9). Additional file 3: Figure S10 (*N* = 20 and *p* = 100), Additional file 3: Figure S11 (*N* = 40 and *p* = 16), and Additional file 3: Figure S12 (*N* = 40 and *p* = 100) demonstrate a similar pattern with even more insignificant differences.

To summarize, Figures 2 and 3 demonstrate, that when a gene set has only a few differentially expressed genes (*γ* = 1/8), edgeR (with GM or FM) has a higher power to detect very small fold changes than the other multivariate and gene-level GSA methods. However, when *γ* = 1/4 and *γ* = 1/2, ROAST has the same power as edgeR with GM or FM. It should be noted that the higher power of edgeR with GM or FM is caused by the higher Type I error of edgeR with GM or FM (Table 2 and see below).

### The analysis of the Nigerian dataset

#### Type I error rate

To estimate how different tests control the Type I error rate for the real data, we performed intra-condition comparisons using only male samples from the Nigerian dataset. The male samples were randomly distributed over two groups, and GSA was conducted using all tests over C2 pathways from the MSigDB [43] database. There should be no gene sets differentially expressed between these two groups. The Type I error rate was averaged over 100 sample permutations (Table 3). For multivariate tests, ROAST has the lowest average Type I error rate, followed by *N*-statistic, KS and WW. Similar to the simulated data when the sample size is large, for real data TMM and QQN normalizations have lower average Type I errors than RPKM and VOOM.

**Table 3:** **Average type I error rates attained from Nigerian male samples, α = 0.05**

	**RPKM**	**QQN**		**TMM**	**VOOM**
N-stat	0.049	0.045		0.044	0.055
WW	0.069	0.062		0.058	0.072
KS	0.052	0.052		0.048	0.059
ROAST					0.033
			FM	SM	GM
edgeR			0.075	0.062	0.119
DESeq			0.068	0.059	0.103
eBayes			0.059	0.057	0.063

Interestingly, for gene-level GSA tests with different *P*-values transformations (FM, SM, GM), the Type I error rate estimates on real data mimic exactly the Type I error rate estimates on simulated data (Tables 1 and 2). All three tests (edgeR, DESeq, and eBayes) that apply GM show the highest Type I error followed by tests with FM and SM respectively. Under each *P*-value’s combining method, edgeR has the highest Type I error rate, followed by DESeq and eBayes.

The Type I error rate estimates on real and simulated data are perfectly correlated for gene-level GSA tests. For real data and multivariate tests, TMM and QQN normalizations lead to the more conservative Type I error rate estimates.

#### Detected pathways

While, for real data, the Type I error rate of different GSA approaches can be directly evaluated by using two subsets from the same group, there is no straightforward and unbiased way to evaluate their power. We selected the Nigerian dataset [42] because it contains two sets of True Positives: genes that are escaping X-chromosome inactivation and are therefore overexpressed in females (XiE), and genes that are located on male-specific region of Y chromosome and are therefore overexpressed in males (msY). All tests detect msY, XiE, and DEX (C2 pathway, containing X-linked genes escaping inactivation) with high significance. All tests fail to detect Xi (all X-linked genes that are not escaping inactivation) except for the univariate tests with GM, because univariate tests with GM have the highest Type I error rate (see Additional file 1: Table S3).

Except for pathways containing gender-specific genes, there is no set of pathways that are guaranteed to be differentially expressed between male and female samples. We therefore decided to examine the entire set of C2 pathways with the goal to quantitatively characterize different methods based on: (1) a number of detected pathways at the different significance levels; (2) the average number of genes in detected pathways; (3) the average length of genes in detected pathways; and (4) the percentage of differentially expressed genes in detected pathways. This information will clarify whether there are methods that are: (1) overlay liberal (detect too many pathways that are not shared with the majority of the other approaches); (2) biased in terms of the number of genes in detected pathways; (3) biased in terms of the average gene length in detected pathways; or (4) detecting only pathways with small (large) number of differentially expressed genes.

Among multivariate tests WW is the most liberal (343 with RPKM, 352 with QQN, 333 with TMM and 348 with VOOM). KS is the next most liberal (267 with RPKM, 292 with QNN, 278 with TMM and 271 with VOOM). *N*-statistic is more conservative than both WW and KS (241 with RPKM, 254 with QNN, 252 with TMM and 245 with VOOM). ROAST is the most conservative among multivariate tests (199 pathways). Methods with QQN normalization detect slightly more pathways as compared to the same method with other normalizations.

Univariate tests with GM detect by far the highest number of pathways (603 with edgeR, 565 with DESeq, and 465 with eBayes). Tests with SM are the most conservative among methods (63 with edgeR, 56 with DESeq, and 77 with eBayes), followed by FM (151 with edgeR, 162 with DESeq, and 146 with eBayes). These observations are in agreement with the Type I error rate estimates for univariate tests with different approaches for combining *P*-values (Tables 2 and 3).

**Figure 4:**
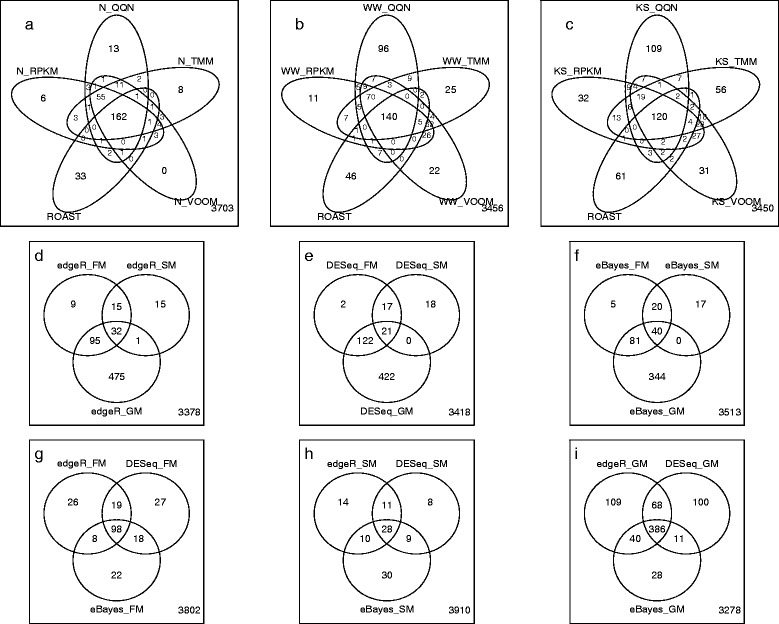
**Venn diagrams showing the number of common pathways detected in the processed Nigerian dataset by multivariate tests with normalizations and univariate tests with combined** ***P*** **-** **values for gene** **-** **level GSA methods (α = 0.05). (a)** N-statistic with different normalizations and ROAST; **(b)** WW with different normalizations and ROAST; **(c)** KS with different normalizations and ROAST; **(d)** edgeR with different *P*-values combining methods; **(e)** DESeq with different *P*-values combining methods; **(f)** eBayes with different *P*-values combining methods; **(g)** univariate tests with FM; **(h)** univariate tests with SM; **(i)** univariate tests with GM.

The Venn diagrams in Figure 4 show the common pathways detected (*α* = 0.05) by multivariate tests with different normalizations (except ROAST which uses VOOM followed by RPKM only) and univariate tests with different *P*-values combining approaches. *N*-statistic detects more common pathways with ROAST than WW and KS and also has more common pathways across different normalizations (Figure 4a). Both WW and KS have much more unique pathways detected by one normalization method than *N*-statistic (Figure 4b,c). When *α* = 0.001 only highly significant pathways are detected, consequently, WW and KS now show similar common groups with ROAST (Additional file 3: Figure S13).

Univariate tests with different *P*-values combining methods have very small overlap between pathways detected by different approaches (Figure 4d,e,f), as compared to multivariate tests. The overlap between pathways detected by different univariate tests with the same *P*-value combining method (Figure 4g,h,i) is larger than the overlap between pathways detected by the same univariate test with different methods for combining *P*-values (Figure 4d,e,f). This demonstrates that the *P*-value combining method is the more important factor than the test itself in detecting DE pathways.

**Figure 5:**
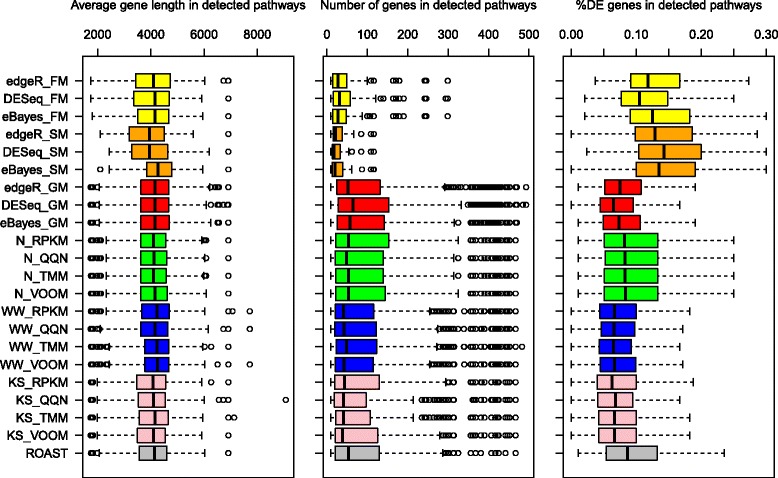
**The percentage of DE genes** **,** **number of genes and average gene length in detected pathways in the processed Nigerian dataset by different methods.**

Figure 5 shows the number of genes, the percentage of DE genes, and the average gene length in detected pathways for all tests (*α* = 0.05). The DE genes in each pathway were found using eBayes. Figure 5 confirms the presence of biases in gene-level tests for GSA that are introduced by different *P*-value combining approaches. Tests with SM and FM favor pathways with a small number of genes and require larger percentages of DE genes in order to detect a pathway. On the contrary tests with GM favor pathways with a large number of genes and require less percentage of DE genes to detect a pathway.

To test whether there are methods that detect pathways with average gene lengths significantly different than the average gene length of all 4020 C2 pathways, Wilcoxon’s non-parametric test was applied. All univariate tests with GM, eBayes with SM, and WW with any normalization, detect pathways with longer genes than the average. The deviation found in eBayes with SM can be attributed to a small number of detected pathways with a small number of genes, which doesn’t allow accurate estimation of the average gene length per detected pathways. On the other hand, tests with GM detect a large number of pathways with a large number of genes, and it also makes the estimate of the average gene length per detected pathway biased.

## Discussion

Here we have presented a comparative power and Type I error rate analyses for self-contained GSA approaches that could be possibly used for RNA-Seq data. In contrast to microarrays, RNA-Seq data consists of discrete counts, therefore GSA approaches developed for microarrays are not directly applicable to RNA-Seq. We have evaluated and compared three multivariate non-parametric approaches (*N*-statistic, Kolmogorov-Smirnov, and Wald-Wolfowitz tests) in combination with four different normalizations (RPKM, TMM, QNN, and VOOM), ROAST, [29] and gene-level GSA methods that use univariate RNA-Seq specific tests (edgeR, DESeq, and eBayes) and employed different methods for combining *P*-values (FM, SM, and GM). In sum we analyzed the performance of twenty-two combinations of tests, including normalization and *P*-value combining methods in the analysis of RNA-Seq data. All approaches were evaluated on simulated and real data, and their significance was evaluated from sample permutations.

We found that for simulated data the Type I error rate and the power of different multivariate approaches in combination with four different normalizations were virtually unaffected by different normalizations. It should be noted that the Type I error rate was only slightly (in the range of 0.01 for the same multivariate test) affected by the normalization used, while the power was not affected at all. Expectedly, both measures were seriously affected by different test statistics. The best-performing approach, in terms of the smallest Type I error rate and the largest power, when the percentage of truly DE expressed genes in a pathway (γ) and a fold change (FC) were small, was ROAST [29], closely followed by *N*-statistic. Multivariate non-parametric Wald-Wolfowitz and Kolmogorov-Smirnov had the smallest power and the largest Type I error rates with all normalizations. The Type I error rate estimates on real data reproduced the trends observed on simulated data. Again, ROAST was the most conservative approach among multivariate tests, and different normalizations didn’t affect the Type I error rates as much as the different test statistics. All of the tests were able to detect gender-specific pathways (msY, XiE and DEX) as differentially expressed between male and female samples with high significance. Xi was not detected by any test.

We also examined the entire set of C2 pathways to quantitatively characterize different methods. The analysis of all C2 pathways confirmed that ROAST is the most conservative among multivariate tests, having the least amount of DE pathways detected. Similarly to the simulated data, ROAST was closely followed by *N*-statistic. Again, for real data, only multivariate test statistics and not normalizations influenced the results to a measurable extent. Thus, on simulated and real data, in terms of the Type I error rate and power, ROAST and *N*-statistic outperformed all other tests, independently of the normalization used. We did not find any evidence of bias for multivariate tests in terms of the number of genes, or the percentage of DE genes in detected pathways with any type of normalization. Surprisingly, among all multivariate tests, multivariate non-parametric Wald-Wolfowitz with any normalization detected pathways with longer genes than the average. It might be related to the fact that WW was the most liberal test among all multivariate tests considered.

For the simulated data, gene-level tests for GSA were heavily dependent on the method used for combining *P*-values, and the differences in power and Type I error rate between univariate tests with the same approach for combining *P*-values were much smaller than the differences when the same test, but different combining *P*-values approaches, were applied. When the percentage of truly differentially expressed genes (*γ*) and fold changes were small, all three tests (edgeR, DESeq, and eBayes) with GM outperformed tests with FM and SM. This difference disappeared when *γ* increased.

For gene-level tests for GSA, it appeared that trends in Type I error rates, estimated from real data, were again similar to the trends in simulated data. All gene-level tests for GSA detected gender-specific pathways, but, in addition, all tests with GM detected the Xi pathway that should not be detected. For gene-level tests for GSA, the analysis of all C2 pathways shows that all of them (except tests with GM) have very small overlap between pathways detected by different approaches as compared to multivariate tests. The overlap between pathways detected by different univariate tests with the same method for combining *P*-values was larger than the overlap between pathways detected by the same univariate test with different methods for combining *P*-values, but still in an order of magnitude smaller than for multivariate tests (excluding tests with GM, see below). This indicates that, first, the *P*-values combining method is the leading factor in detecting DE pathways using gene-level tests for GSA, and, second, for real data they have less power than multivariate approaches in an order of magnitude.

The analysis of C2 pathways on the Nigerian data confirmed our expectations, which were formed by the analysis of the functional dependencies between the original and transformed *P*-values for different *P*-values combining methods (Figure 1). All tests with GM exclusively detected pathways with a large number of genes and a small percentage of DE genes as compared to the other approaches. All tests with SM exclusively detected pathways with a small number of genes and a large percentage of DE genes as compared to the other approaches. The Type I error rate, the number of genes and the percentage of DE genes necessary to detect a pathway for all tests with FM, were exactly in-between GM and SM: smaller than for all tests with GM and larger than for all tests with SM. In agreement tests with GM and eBayes with SM all detected pathways with longer genes than the average (Wilcoxon’s test, Figure 5).

The results from simulated and real data show that gene-level tests for GSA with GM have the highest Type I error rates and the highest power. In addition all tests with GM had the highest number of genes and the smallest percentage of truly DE genes in detected C2 pathways. These observations indicate that the gain in power for tests with GM is caused by the gain in false positives. Tests with SM had the smallest power and the smallest Type I error rates, while the results for tests with FM were intermediate.

It should be noted that recently edgeR with GM was found to outperform many other approaches for GSA in terms of power and Type I error rate and was recommended for RNA-Seq data analysis [25]. Indeed, we observed that edgeR with GM had the highest power among all the other approaches. In a recent publication edgeR with GM was suggested to be the first method of choice for GSA of RNA-Seq data [25]. In contrast to this result, our study showed that for simulated and real data edgeR with GM has the highest Type I error rate among all the other tests for GSA. We hypothesize that the difference between the two studies stems from the way the data were simulated. In our study we used the Negative Binomial model, which is used in edgeR for finding DE genes. In [25] the multivariate normal distribution with fixed correlation structure was used, but, surprisingly, edgeR was used for finding DE genes. Therefore, in the latter case, the distributional assumption of the method (edgeR) was not met, which could have led to the bias in the estimation of the Type I error rates. However, all simulations are only crude approximations of biological reality. To estimate the Type I error rates on the real data, we performed intra-condition comparisons using only male samples from the Nigerian dataset: there should not be gene sets differentially expressed between these two groups. Again, edgeR with GM had the highest Type I error rate for real data among all other tests, confirming that in contrast with [25] results, edgeR with GM has inadequate control of the Type I error rate.

## Conclusions

Overall, for the self-contained category of GSA, multivariate GSA tests are insensitive to different normalizations and have better control of Type I error rates and higher power as compared to gene-level GSA tests, both on simulated and real data. In addition, while standard gene set over-representation analysis shows as over-represented categories with longer genes [19], standard multivariate GSA tests (except WW) with different normalizations do not have any biases in terms of the pathway size, the percentage of DE genes, or the average gene length in a pathway. The opposite is true for all gene-level GSA tests. Thus, our study argues against the use of gene-level tests for GSA whether with Fisher’s combining probabilities Method [27], or Stouffer’s Method [28], or the soft thresholding Gamma Method [25], and emphasize the importance of using non-parametric multivariate tests for detecting DE pathways for RNA-Seq data.

### Availability of software

Software implementing the multivariate generalizations of the Kolmogorov-Smirnov and Wald-Wolfowitz tests in R was released within the GSAR package in version 3.0 of Bioconductor (http://www.bioconductor.org/packages/release/bioc/html/GSAR.html).

## Additional files

Additional file 1: **Supplementary material containing background, details of data processing steps and Supplementary Tables S1-S3.**

Additional file 2: **Supplementary lists of all the genes and their descriptions in msY, XiE, DEX and Xi gene sets.**

Additional file 3: **Supplementary figures containing Supplementary figures S1-S13.**

## Abbreviations

DE: Differentially expressed
GSA: Gene set analysis
GSEA: Gene set enrichment analysis
WW: Wald-Wolfowitz
KS: Kolmogorov-Smirnov
ROAST: Rotation gene set test
FC: Fold change
FM: Fisher’s method
GM: Gamma method
SM: Stouffer’s method
STT: Soft truncation threshold
RPKM: Reads per kilobase per million
QQN: Quantile-quantile normalization
TMM: Trimmed mean of M-values
NB: Negative bionomial
msY: male specific genes of chromosome Y
XiE: Chromosome X genes inactivation escaping
DEX: Disteche escaped from chromosome X inactivation
MSigDB: Molecular signature database
GO: Gene Ontology
KEGG: Kyoto Encyclopedia of genes and genomes

## References

[1] Core LJ, Waterfall JJ, Lis JT (2008). Nascent RNA sequencing reveals widespread pausing and divergent initiation at human promoters. Science.

[2] Wilhelm BT, Marguerat S, Watt S, Schubert F, Wood V, Goodhead I, Penkett CJ, Rogers J, Bahler J (2008). Dynamic repertoire of a eukaryotic transcriptome surveyed at single-nucleotide resolution. Nature.

[3] Robinson MD, McCarthy DJ, Smyth GK (2010). **edgeR**: **a Bioconductor package for differential expression analysis of digital gene expression data**. Bioinformatics.

[4] Anders S, Huber W (2010). Differential expression analysis for sequence count data. Genome Biol.

[5] Li J, Tibshirani R (2013). Finding consistent patterns: A nonparametric approach for identifying differential expression in RNA-Seq data. Stat Methods Med Res.

[6] Smyth G, Smyth G, Gentleman R, Carey V, Dudoit S, Irizarry R, Huber W (2005). **Limma**: **linear models for microarray data**. Bioinformatics and Computational Biology Solutions using R and Bioconductor.

[7] Law CW, Chen Y, Shi W, Smyth GK (2014). Voom: precision weights unlock linear model analysis tools for RNA-seq read counts. Genome Biol.

[8] Mootha VK, Lindgren CM, Eriksson KF, Subramanian A, Sihag S, Lehar J, Puigserver P, Carlsson E, Ridderstrale M, Laurila E, Houstis N, Daly MJ, Patterson N, Mesirov JP, Golub TR, Tamayo P, Spiegelman B, Lander ES, Hirschhorn JN, Altshuler D, Groop LC (2003). PGC-1alpha-responsive genes involved in oxidative phosphorylation are coordinately downregulated in human diabetes. Nat Genet.

[9] Glazko GV, Emmert-Streib F (2009). Unite and conquer: univariate and multivariate approaches for finding differentially expressed gene sets. Bioinformatics.

[10] Emmert-Streib F, Glazko GV (2011). Pathway analysis of expression data: deciphering functional building blocks of complex diseases. PLoS Comput Biol.

[11] Ackermann M, Strimmer K (2009). A general modular framework for gene set enrichment analysis. BMC Bioinformatics.

[12] da Huang W, Sherman BT, Lempicki RA (2009). Bioinformatics enrichment tools: paths toward the comprehensive functional analysis of large gene lists. Nucleic Acids Res.

[13] Dinu I, Potter JD, Mueller T, Liu Q, Adewale AJ, Jhangri GS, Einecke G, Famulski KS, Halloran P, Yasui Y (2009). Gene-set analysis and reduction. Brief Bioinform.

[14] Goeman JJ, Buhlmann P (2007). Analyzing gene expression data in terms of gene sets: methodological issues. Bioinformatics.

[15] Tian L, Greenberg SA, Kong SW, Altschuler J, Kohane IS, Park PJ (2005). Discovering statistically significant pathways in expression profiling studies. Proc Natl Acad Sci USA.

[16] da Huang W, Sherman BT, Lempicki RA (2009). Systematic and integrative analysis of large gene lists using DAVID bioinformatics resources. Nat Protoc.

[17] Khatri P, Sirota M, Butte AJ (2012). Ten years of pathway analysis: current approaches and outstanding challenges. PLoS Comput Biol.

[18] Rahmatallah Y, Emmert-Streib F, Glazko G (2012). Gene set analysis for self-contained tests: complex null and specific alternative hypotheses. Bioinformatics.

[19] Young MD, Wakefield MJ, Smyth GK, Oshlack A (2010). Gene ontology analysis for RNA-seq: accounting for selection bias. Genome Biol.

[20] Hanzelmann S, Castelo R, Guinney J (2013). GSVA: gene set variation analysis for microarray and RNA-seq data. BMC Bioinformatics.

[21] Wang X, Cairns MJ (2013). **Gene set enrichment analysis of RNA**-**Seq data**: **integrating differential expression and splicing**. BMC Bioinformatics.

[22] Tripathi S, Glazko GV, Emmert-Streib F (2013). Ensuring the statistical soundness of competitive gene set approaches: gene filtering and genome-scale coverage are essential. Nucleic Acids Res.

[23] Varemo L, Nielsen J, Nookaew I (2013). Enriching the gene set analysis of genome-wide data by incorporating directionality of gene expression and combining statistical hypotheses and methods. Nucleic Acids Res.

[24] Dinu I, Potter JD, Mueller T, Liu Q, Adewale AJ, Jhangri GS, Einecke G, Famulski KS, Halloran P, Yasui Y (2007). Improving gene set analysis of microarray data by SAM-GS. BMC Bioinformatics.

[25] Fridley BL, Jenkins GD, Grill DE, Kennedy RB, Poland GA, Oberg AL (2013). Soft truncation thresholding for gene set analysis of RNA-seq data: application to a vaccine study. Sci Rep.

[26] Friedman JH, Rafsky C (1979). Multivariate Generalizations of the Wald-Wolfowitz and Smirnov Two-Sample Tests. Ann Stat.

[27] Fisher R (1932). Statistical methods for research workers.

[28] Stouffer S, DeVinney L, Suchmen E (1949). The American Soldier: Adjustment during army life., vol. 1.

[29] Wu D, Lim E, Vaillant F, Asselin-Labat ML, Visvader JE, Smyth GK (2010). ROAST: rotation gene set tests for complex microarray experiments. Bioinformatics.

[30] Baringhaus L, Franz C (2004). On a new multivariate two-sample test. J Multivariate Anal.

[31] Klebanov L, Glazko G, Salzman P, Yakovlev A, Xiao Y (2007). A multivariate extension of the gene set enrichment analysis. J Bioinform Comput Biol.

[32] Zaykin DV (2011). Optimally weighted Z-test is a powerful method for combining probabilities in meta-analysis. J Evol Biol.

[33] Zaykin DV, Zhivotovsky LA, Czika W, Shao S, Wolfinger RD (2007). Combining p-values in large-scale genomics experiments. Pharm Stat.

[34] Quackenbush J (2002). Microarray data normalization and transformation. Nat Genet.

[35] Irizarry RA, Hobbs B, Collin F, Beazer-Barclay YD, Antonellis KJ, Scherf U, Speed TP (2003). Exploration, normalization, and summaries of high density oligonucleotide array probe level data. Biostatistics.

[36] Mortazavi A, Williams BA, McCue K, Schaeffer L, Wold B (2008). Mapping and quantifying mammalian transcriptomes by RNA-Seq. Nat Methods.

[37] Dillies MA, Rau A, Aubert J, Hennequet-Antier C, Jeanmougin M, Servant N, Keime C, Marot G, Castel D, Estelle J, Guernec G, Jagla B, Jouneau L, Laloë D, Le Gall C, Schaëffer B, Le Crom S, Guedj M, Jaffrézic F, French StatOmique Consortium (2012). A comprehensive evaluation of normalization methods for Illumina high-throughput RNA sequencing data analysis. Brief Bioinform.

[38] Oshlack A, Wakefield MJ (2009). Transcript length bias in RNA-seq data confounds systems biology. Biol Direct.

[39] Robinson MD, Oshlack A (2010). A scaling normalization method for differential expression analysis of RNA-seq data. Genome Biol.

[40] Bullard JH, Purdom E, Hansen KD, Dudoit S (2010). Evaluation of statistical methods for normalization and differential expression in mRNA-Seq experiments. BMC Bioinformatics.

[41] Hansen KD, Irizarry RA, Wu Z (2012). Removing technical variability in RNA-seq data using conditional quantile normalization. Biostatistics.

[42] Pickrell JK, Marioni JC, Pai AA, Degner JF, Engelhardt BE, Nkadori E, Veyrieras JB, Stephens M, Gilad Y, Pritchard JK (2010). Understanding mechanisms underlying human gene expression variation with RNA sequencing. Nature.

[43] Liberzon A, Subramanian A, Pinchback R, Thorvaldsdottir H, Tamayo P, Mesirov JP (2011). Molecular signatures database (MSigDB) 3.0. Bioinformatics.

[44] Disteche CM, Filippova GN, Tsuchiya KD (2002). Escape from X inactivation. Cytogenet Genome Res.

[45] Robinson MD, Smyth GK (2007). Moderated statistical tests for assessing differences in tag abundance. Bioinformatics.

