# Supplementary material for: Comparative evaluation of gene set analysis approaches for RNA-Seq data
Source: BMC Bioinformatics. 2014 Dec 5;15(1):397. doi: 10.1186/s12859-014-0397-8 (PMC4265362; doi:10.1186/s12859-014-0397-8)
Supplement: Supplementary file 1 — Supplementary material containing background, details of data processing steps and Supplementary Tables S1-S3. [file 12859_2014_397_MOESM1_ESM.pdf]

Comparative evaluation of gene set analysis approaches for RNA-Seq data

Yasir Rahmatallah[1], Frank Emmert-Streib[2] and Galina Glazko[1,*]
[1]Division of Biomedical Informatics, University of Arkansas for Medical Sciences, Little Rock, AR 72205.
[2]Computational Biology and Machine Learning Laboratory, Center for Cancer Research and Cell Biology, School of Medicine, Dentistry and Biomedical Sciences, Queen's University Belfast, 97 Lisburn Road, Belfast, BT9 7BL, UK.

ABSTRACT

This document provides supplementary material regarding the dataset and methods used in the main manuscript including univariate test statistics, *P*-values combining methods and normalization methods. Details of preprocessing steps of the Nigerian dataset in addition to obtaining the C2 pathways set from the molecular signature database and the gender-specific genes are also presented. Finally, the simulation setup for generating random counts useful to test the performance of different methods is presented.

Univariate Tests

We used three popular parametric univariate tests to detect differentially expressed (DE) genes between two phenotypes: edgeR [1], DESeq [2] and eBayes [3]. The obtained gene-level P-values using these tests are combined into gene set P-values using three different approaches: Fisher's combining probabilities Method (FM) [4], Stouffer's Method (SM) [5] and the soft thresholding Gamma Method (GM) [6].

edgeR

This test was developed using the Negative Binomial (NB) distribution to model the gene-level dispersions in count data and uses conditional weighted likelihood estimator to shrink individual dispersions towards a common dispersion [1]. The amount of shrinkage is selected according to an approximate empirical Bayes rule. Accurate estimation of dispersion for NB data is critical for assessing the significance of changes in the mean between experimental conditions. The test is suitable for small counts and can make use of minimal number of replicates. The test is implemented in the Bioconductor package edgeR [7].

DESeq

This test also uses the NB distribution to model the count data and is based on the assumption that most genes are non-DE. The variance σ_{ij}^2 of the counts observed for gene i in sample j is modeled as

$$\sigma_{ij}^2 = \mu_{ij} + s_j^2 v_{i,\rho(j)}$$

where μ_{ij} is the expectation value of the observed counts for gene i in sample j, s_j is the library size factor, $v_{i,\rho(j)}$ is the raw variance and $\rho(j)$ is the experimental condition for sample j. The test uses the median of the ratios of sample counts to those of a pseudo reference sample as the library size factor for each sample to normalize against differences in library sizes. The pseudo reference sample is obtained by taking the geometric mean across samples. The test uses a generalized linear model of the gamma family for the local regression used to estimate raw variance [2]. This test is implemented in the Bioconductor package DESeq.

eBayes

This test fits linear models for each gene in the dataset and uses an empirical Bayes shrinkage method to moderate the standard errors of the estimated log-fold changes towards a common value. For assessing differential expression, a moderated t-statistic is used. Smoothing the standard errors has the effect of borrowing information among genes to aid with each gene's inference. The moderated t-statistic has higher degrees of freedom compared to the ordinary t-statistic, reflecting the greater reliability associated with the smoothed standard errors [3]. This test is implemented in the Bioconductor package limma.

Combining *P*-values

In the context of GSA, gene-level P-values can be aggregated in order to provide a combined P-value for the overall hypothesis applied to a gene set. This approach agrees with the aim of GSA to detect a gene set when multiple genes have small or moderate effects. It reduces the multiple testing burden and incorporates biological knowledge to increase the power to detect meaningful associations. Most of the differential analysis methods for count data produce a significance measure (P-value) by testing hypotheses at the gene level. In order to study the effects of combining the P-values obtained by different tests to

* Correspondence: gvglazko@uams.edu

perform GSA, we examined three common combining methods: Fisher combined probability (Fisher's method) [4], normal inverse (Stouffer's method) [5] and soft truncation threshold Gamma Method (GM) [6]. These methods perform the same general procedure, where P-values obtained by testing L hypotheses at the gene level are transformed by some function H and then summed (possibly using gene-specific weights w_is) to calculate the combined test statistic, $T = \sum_{i=1}^{L} w_i H(P_i)$, where L is the number of genes in the gene set. If weights are not used, we set all w_is to $1/L$. The combined P-value P_c is computed using the distribution of the combined test statistic T. However, due to the lack of independence between genes, such parametric approach is rendered inaccurate. Instead, we estimate the null distribution of T using a non-parametric approach based on random permutations of sample labels to preserve the correlation structure between genes and calculate the gene-level P-values and T for 1000 permutation to get a reasonable estimate of the null distribution. The empirical estimate of the combined P-value P_c is the proportion of the permutations in which T was found to be larger than the observed T of the gene set. It is worth stating here that no combining method is uniformly most powerful under all conditions.

Fisher's Method (FM)

This method transforms P-values using the natural logarithm and the combined test statistic is given by

$$T = -2\sum_{i=1}^{L} w_i \, log_e(P_i) = -2\sum_{i=1}^{L}\frac{1}{L} log_e(P_i) = \frac{-2}{L}\, log_e\left(\prod_{i=1}^{L} P_i\right)$$

When all L null hypotheses are true and independent, T follows a chi-square distribution with $2L$ degrees of freedom [4]. When L is very large, this test statistic favors P-values smaller than $1/e \approx 0.368$ such that

$$P_c \to \begin{cases} 0 & P_i < 1/e\,, \forall i \\ 1 & P_i > 1/e\,, \forall i \\ 1/e & P_i = 1/e\,, \forall i \end{cases}$$

However, this threshold point is a little smaller than $1/e$ for a finite value of L.

Stouffer's Method (SM)

This method transforms P-values into standard normal scores (Z-scores) using the inverse standard normal cumulative distribution function such that $Z_i = \Phi^{-1}(1-P_i)$ and combine them (possibly using weights w_is) into the combined test statistic

$$T = \frac{\sum_{i=1}^{L} w_i Z_i}{\sqrt{\sum_{i=1}^{L} w_i^2}}$$

which follows a standard normal distribution when all L null hypotheses are true and independent [5]. This test statistic favors P-values smaller than 0.5.

Gamma Method (GM)

This method is based on summing the transformed gene-level P-values using an inverse gamma cumulative distribution function $G_{w,1}^{-1}$ where w is the shape parameter, i.e. the combined test statistic is given by [8]

$$T = \sum_{i=1}^{L} G_{w,1}^{-1}(1-P_i)$$

This combining method exploits the feature that the sum of gamma distributed random variables with the same rate has a gamma distribution [8]. The shape parameter w controls the amount of emphasis given to gene-level P-values below a particular threshold. This feature is imposed by any transformation function H and is referred to as soft truncation threshold (STT) [6, 8]. It is useful when there is pronounced heterogeneity in effects. The STT is controlled by w such that $w = G_{w,1}^{-1}(1-STT)$. When w is large, GM becomes equivalent to the inverse normal Stouffer's method which has $STT = 0.5$ and when it is 1, it becomes equivalent to Fisher's method with $STT = 1/e$. Fridley [6] examined the performance of GM with various STT values and recommended that generally STT values between 0.01 and 0.36 tend to give the best power. For our work, we chose $w = 0.0137$ which gives $STT = 0.05$.

Normalization methods

Technical biases in RNA-Seq experiments include between-sample differences such as the total number of reads per sample or library size [9] as well as within-sample variations related to gene length [10] and, to a lesser degree, the percent of C or G nucleotides in a gene or GC-content [11]. Therefore, raw counts are neither directly comparable between genes within one sample, nor between samples for the same gene. The counts of each gene are expected to be proportional to both gene abundance (molar concentration) and gene length as longer genes are expected to produce more reads in the sequencing process. The counts will also vary between samples as a result of differences in the total number of mapped reads per sample (library size or sequencing depth). To allow direct comparison between gene sets in the context of differential gene set analysis, proper normalizations must be used [12]. We adopted four popular normalization strategies to examine the performance of our multivariate tests.

Reads per kilobase per million (RPKM)

The reads per kilobase per million (RPKM) mapped reads measure normalizes the raw gene counts by the total number of mapped reads in each sample and the gene lengths [9]. It is a popular normalization procedure for count data. The RPKM for the number of reads mapped to gene g, n_g, which has a gene length L_g is calculated as

$$RPKM_g = \frac{n_g \times 10^9}{L_g \times N}$$

where N is the total number of mapped reads in the sample (sequencing depth). This normalization facilitates the comparison of transcript levels both between and within samples. The implementation of the RPKM normalization is available in the Bioconductor package edgeR.

Quantile-quantile normalization (QQN)

The quantile-quantile normalization (QQN) method was first proposed in the context of microarray data to match the distributions of probe level intensities across arrays and reduce between-sample differences [13]. In the context of differential gene set analysis for RNA-Seq count data, we combine QQN with RPKM to account for differences in gene lengths: First, the QQN normalization matches the distributions of gene counts across samples; Second, RPKM normalizes by gene lengths and sequencing depth. Since the differences between samples in sequencing depth were mitigated by QQN, RPKM here mainly normalizes against gene length differences. Both steps can be implemented using the functions available in the Bioconductor package edgeR.

Trimmed mean of M-values (TMM)

The trimmed mean of M-values (TMM) normalization method is an empirical strategy that equates the overall expression levels of genes between samples under the assumption that the majority of genes are not differentially expressed [14]. It estimates a normalization factor using a weighted trimmed mean of the log expression ratio between the samples and one selected reference sample. To provide a robust estimation, the upper and lower values of the log expression ratio and absolute expression levels are removed before calculating the weighted average. After trimming, a weighted mean of the log expression ratio is calculated, with weights as the inverse of the approximate asymptotic variances [14]. According to the hypothesis of few DE genes, TMM should be close to 1. When the hypothesis is false (TMM≠1), TMM value provides an estimate of the normalization factor that must be applied to the raw counts in each sample to fulfil the hypothesis. The TMM normalization is implemented in the Bioconductor package edgeR. Since TMM was suggested for between-samples normalization only, we follow it by RPKM to normalize against gene length differences.

VOOM

The VOOM normalization was motivated by the idea of applying normal-based microarray-like statistical methods to RNA-Seq count data, with the idea that it is more important to model the mean-variance trend correctly than it is to determine the exact distribution of the counts [15]. This method starts by fitting gene-level linear models to the normalized logged counts per million (CPM) of the raw counts. Then, a robust trend fits the generated residual standard deviations by the linear models as a function of the average log-CPM. Taking the total number of counts per observation into account, the fitted log-CPM for each observation is converted into a predicted count. The mean-variance trend is incorporated to predict the variance of each log-CPM observation based on its predicted count size. Then, the inverse predicted variance for each observation is incorporated as an associated weight into the linear model to eliminate the mean-variance relationship [15]. This feature makes VOOM especially suited for the rotation gene set test (ROAST) [16].

Since VOOM achieves between-samples normalization only, we follow it by RPKM normalization to account for gene length differences. This combination makes VOOM suitable for ROAST and other proposed multivariate methods in the context of GSA. Since VOOM returns normalized data in log scale, we transform the data back to linear scale before applying the RPKM normalization.

Preprocessing the Nigerian dataset

In this study, we relied on a subset of the Pickrell [11] dataset of sequenced cDNA libraries generated from 69 lymphoblastoid cell lines derived from Yoruban Nigerian individuals (YRI) as part of the HapMap project. Raw unmapped RNA-Seq short reads in fastq format files are available at [17]. Only samples sequenced at the Yale sequencing center using the Illumina Genome Analyzer 2 platform were used here. Among adult-and-both-parents trios available, we considered only 58 unrelated individual (parents) with 29 males and 29 females. The raw 35 base pairs (bp) short reads were aligned to the UCSC hg19 human genome using the Bowtie aligner [18] with no mismatches allowed. Supplementary Table S1 shows the numbers and percentages of short reads aligned by Bowtie and mapped uniquely to an annotated gene. The lists of genomic features (exons) categorized by genes (with ENTREZ identifiers) under the hg19 human genome model were obtained using the Bioconductor GenomicFeatures package (version 1.12.4). Gene counts were obtained by detecting the overlaps between short reads and the list of genomic ranges under each gene using the Bioconductor GenomicRanges package (version 1.12.5). Short reads which have non-unique mappings were discarded. The obtained count matrix had short reads mapped to a total of 23754 annotated genes with a total number of mapped reads (counts) per sample ranging between 0.885×10^6 and 3.612×10^6.
To ensure only expressed genes are considered further, we filter out any gene which does not satisfy any of two conditions: have nonzero counts in more than 20 samples out of the total 58; and have a mean counts per million (cpm) greater than 1. Gene length information with ENTREZ identifiers were obtained from the Bioconductor GSVAdata package (version

0.99.11). Any genes with no supported gene length information were discarded. Finally, genes' ENTREZ identifiers were replaced by unique SYMBOL identifiers using the Bioconductor org.Hs.eg.db annotation package (version 2.10.1). The resulted count matrix has a total of 13191 annotated genes and 58 samples (29 males and 29 females) with total counts ranging between 0.875×10^6 and 3.594×10^6. After different normalizations are used, the normalized counts Y_{ij} are transformed to log-scale using the function $\log_2\left(1+Y_{ij}\right)$ to further reduce the effects of outliers.

C2 and Gender-Related Pathways

Gene sets were taken from the C2 pathways set of the molecular signature database (MSigDB) 3.0 [19]. These pathways were obtained using the Bioconductor package GSEABase (version 1.24.0). We discarded any gene identifier not present in the filtered dataset from C2 pathways and considered only pathways with p genes where $10\leq p\leq500$. We also built three special pathways and included them in our GSA. The resulted dataset comprised 12051 genes and 4020 pathways to analyze.

One way to validate the performance of gene set tests while performing GSA for the Nigerian dataset, considering male and female phenotypes, is to test pathways containing gender-specific genes. While such approach is straightforward for gene-level tests, GSA involves testing pathways with different numbers of gender-specific genes. Most C2 pathways contain no or negligible amount of gender-specific genes. Even when a pathway has a few such genes, this doesn't indicate that the pathway should be differentially expressed. For these reasons we chose to examine a few customized pathways. We examined three pathways containing gender-specific genes (hence considered truly DE between males and females) and one additional pathway with genes linked to chromosome X but not related to gender differences (do not escape chromosome X inactivation). The first two gender-related pathways were built from genes located in male-specific region of the Y chromosome (msY), and therefore are over-expressed in males, and genes that escape chromosome X inactivation in female samples (XiE), and therefore are over-expressed in females. The Bioconductor packages tweeDEseqCountData and GSVAdata provide these lists of genes with ENSEMBL and ENTREZ identifiers, respectively. We used the Bioconductor org.Hs.eg.db annotation package (version 2.10.1) to map both ENSEMBL and ENTREZ identifiers into SYMBOL identifiers (considering unique mappings only) and build the two pathways using the union of gene lists from both packages. Genes that were filtered during dataset preprocessing were ignored. Finally, the male-specific Y-linked genes pathway (msY) contained 11 genes and the X-linked genes that escape inactivation (XiE) pathway contained 48 genes (Supplementary Table S2). We also examined the curated C2 pathway DISTECHE_ESCAPED_FROM_X_INACTIVATION (DEX) which bundles 13 X-linked genes found in our filtered dataset and were reported to escape inactivation [20]. From these 13 genes, 11 were also found in XiE. The fourth pathway Xi was built from chromosome X genes that fail to escape inactivation (chromosome X genes excluding XiE) and contained 387 genes from our filtered dataset (see Additional file 2 for the full lists of the genes in msY, XiE, Xi and DES pathways). Unlike pathway Xi, pathways msY, XiE and DES are all expected to be found differentially expressed by any reasonable test. The results of testing these four pathways using all the methods presented in this study are shown in Supplementary Table S3.

Simulation of RNA-Seq Counts

We model the count for gene i in sample j by a random variable Y_{ij} with Negative Binomial (NB) distribution

$$Y_{ij}\sim NB\left(mean=\mu_{ij}, var=\mu_{ij}(1+\mu_{ij}\varphi_{ij})\right)=NB\left(\mu_{ij},\varphi_{ij}\right)$$

where μ_{ij} and φ_{ij} are respectively the mean count and dispersion parameter of gene i in sample j. For each gene in a gene set, a vector of mean count, dispersion and gene length information (μ_i,φ_i,L_i) is randomly selected from a pool of vectors derived from the processed Nigerian dataset. To provide a more uniform set of samples to estimate the mean count and dispersion parameters, we considered genes which have nonzero counts in more than 20 samples out of the total 58, have 1<cpm<1000 and used a subset of 44 samples for which the total number of mapped reads per sample ranged between 1.5×10^6 and 2.5×10^6. The dispersion parameter for each gene was estimated using the Bioconductor package edgeR (version 3.4.2) as follows: First, the total number of counts per each of the 44 samples was reduced to that of the sample with minimum total counts by binomial thinning. Second, the dispersion parameter for each gene is estimated by an empirical Bayes method based on weighted conditional maximum likelihood [1]. The obtained parameters pool from the reduced Nigerian dataset has 13109 vectors (μ_i,φ_i,L_i) from which negative binomial random counts can be generated and later normalized. It is worth mentioning here that the generation of synthetic counts does not require an accurate estimation of μ_i and φ_i for each gene but simply to provide a plausible distribution of values of these parameters representing their typical abundances and variation due to biological and technical effects [21]. After the NB count data is generated, the specific normalization method with each univariate test is used and different normalizations are used with multivariate tests. Finally, normalized counts are transformed to log-scale using the transformation function $\log_2\left(1+Y_{ij}\right)$ to further reduce the effects of outliers. Supplementary Figures S1 and S2 (see Additional file 3) respectively show the density and histogram plots for the original counts of 13109 genes in 44 samples and NB simulated counts with the same number of genes and samples before and after different normalizations. The simulated counts match the original counts reasonably well.

REFERENCES

1. Robinson MD, Smyth GK: **Moderated statistical tests for assessing differences in tag abundance.** *Bioinformatics* 2007, **23:**2881-2887.

2. Anders S, Huber W: **Differential expression analysis for sequence count data.** *Genome biology* 2010, **11:**R106.

3. Smyth GK: **Linear models and empirical Bayes methods for assessing differential expression in microarray experiments.** *Stat Appl Genet Mol Biol* 2004, **3:**3.

4. Fisher R: *Statistical methods for research workers.* Edinburgh, Scotland, Oliver and Boyd; 1932.

5. Stouffer S, DeVinney L, Suchmen E: *The American Soldier: Adjustment during army life.* Princeton, US: Princeton University Press; 1949.

6. Fridley BL, Jenkins GD, Grill DE, Kennedy RB, Poland GA, Oberg AL: **Soft truncation thresholding for gene set analysis of RNA-seq data: application to a vaccine study.** *Sci Rep* 2013, **3:**2898.

7. Robinson MD, McCarthy DJ, Smyth GK: **edgeR: a Bioconductor package for differential expression analysis of digital gene expression data.** *Bioinformatics* 2010, **26:**139-140.

8. Zaykin DV, Zhivotovsky LA, Czika W, Shao S, Wolfinger RD: **Combining p-values in large-scale genomics experiments.** *Pharmaceutical statistics* 2007, **6:**217-226.

9. Mortazavi A, Williams BA, McCue K, Schaeffer L, Wold B: **Mapping and quantifying mammalian transcriptomes by RNA-Seq.** *Nature methods* 2008, **5:**621-628.

10. Oshlack A, Robinson MD, Young MD: **From RNA-seq reads to differential expression results.** *Genome biology* 2010, **11:**220.

11. Pickrell JK, Marioni JC, Pai AA, Degner JF, Engelhardt BE, Nkadori E, Veyrieras JB, Stephens M, Gilad Y, Pritchard JK: **Understanding mechanisms underlying human gene expression variation with RNA sequencing.** *Nature* 2010, **464:**768-772.

12. Dillies MA, Rau A, Aubert J, Hennequet-Antier C, Jeanmougin M, Servant N, Keime C, Marot G, Castel D, Estelle J, et al: **A comprehensive evaluation of normalization methods for Illumina high-throughput RNA sequencing data analysis.** *Briefings in bioinformatics* 2012.

13. Irizarry RA, Hobbs B, Collin F, Beazer-Barclay YD, Antonellis KJ, Scherf U, Speed TP: **Exploration, normalization, and summaries of high density oligonucleotide array probe level data.** *Biostatistics* 2003, **4:**249-264.

14. Robinson MD, Oshlack A: **A scaling normalization method for differential expression analysis of RNA-seq data.** *Genome biology* 2010, **11:**R25.

15. Law CW, Chen Y, Shi W, Smyth GK: **Voom: precision weights unlock linear model analysis tools for RNA-seq read counts.** *Genome Biol* 2014, **15:**R29.

16. Wu D, Lim E, Vaillant F, Asselin-Labat ML, Visvader JE, Smyth GK: **ROAST: rotation gene set tests for complex microarray experiments.** *Bioinformatics* 2010, **26:**2176-2182.

17. **Raw unmapped RNA-Seq short reads in fastq format files.** [http://eqtl.uchicago.edu/RNA_Seq_data/unmapped_reads/]

18. Langmead B, Trapnell C, Pop M, Salzberg SL: **Ultrafast and memory-efficient alignment of short DNA sequences to the human genome.** *Genome Biol* 2009, **10:**R25.

19. Liberzon A, Subramanian A, Pinchback R, Thorvaldsdottir H, Tamayo P, Mesirov JP: **Molecular signatures database (MSigDB) 3.0.** *Bioinformatics* 2011, **27:**1739-1740.

20. Disteche CM, Filippova GN, Tsuchiya KD: **Escape from X inactivation.** *Cytogenet Genome Res* 2002, **99:**36-43.

21. Robles JA, Qureshi SE, Stephen SJ, Wilson SR, Burden CJ, Taylor JM: **Efficient experimental design and analysis strategies for the detection of differential expression using RNA-Sequencing.** *BMC Genomics* 2012, **13:**484.

Table S1. Nigerian Short Reads Alignment Summary

Sample	Nigerian Yale sample (35 bp short reads)	Gender (M/F)	# Reads processed (FASTQ)	# Reported alignments (by Bowtie)	% Reported alignments (by Bowtie)	# Alignments to a unique hg19 gene	% Alignments to a unique hg19 gene
1	NA18501_yale.bam	M	6422000	5838083	90.91	2518755	43.14
2	NA18502_yale.bam	F	8909231	7976026	89.53	3471444	43.52
3	NA18504_yale.bam	M	7113128	6380433	89.70	2711657	42.50
4	NA18505_yale.bam	F	8849477	8057799	91.05	3315166	41.14
5	NA18507_yale.bam	M	10329429	9219847	89.26	3897181	42.27
6	NA18508_yale.bam	F	8701858	7896491	90.74	3384648	42.86
7	NA18516_yale.bam	M	7818182	7158331	91.56	2986097	41.71
8	NA18517_yale.bam	F	9367811	8513394	90.88	3763158	44.20
9	NA18522_yale.bam	M	7691810	6968610	90.60	3080228	44.20
10	NA18523_yale.bam	F	4528349	3796290	83.83	1659570	43.72
11	NA18852_yale.bam	F	9606138	8571651	89.23	3745137	43.69
12	NA18853_yale.bam	M	9717230	8967320	92.28	2909908	32.45
13	NA18855_yale.bam	F	7930075	6943915	87.56	3052765	43.96
14	NA18856_yale.bam	M	5267997	4719944	89.60	1931678	40.93
15	NA18858_yale.bam	F	8800059	7884153	89.59	3429896	43.50
16	NA18859_yale.bam	M	10007505	8750612	87.44	3866788	44.19
17	NA18861_yale.bam	F	4642527	4228902	91.09	1911930	45.21
18	NA18862_yale.bam	M	8619671	7799474	90.48	3115251	39.94
19	NA18870_yale.bam	F	6833330	6079199	88.96	2591506	42.63
20	NA18871_yale.bam	M	6261611	5497420	87.80	2406701	43.78
21	NA18912_yale.bam	F	4211405	3905879	92.75	1581472	40.49
22	NA18913_yale.bam	M	9006101	7960151	88.39	3470580	43.60

23	NA19092_2_yale.bam	M	11123460	9840519	88.47	4403764	44.75
24	NA19093_yale.bam	F	6997219	6342108	90.64	2765276	43.60
25	NA19098_yale.bam	M	10280860	9381273	91.25	4167406	44.42
26	NA19099_yale.bam	F	9955015	8956531	89.97	3873755	43.25
27	NA19101_yale.bam	M	9320429	8497804	91.17	3665691	43.14
28	NA19102_yale.bam	F	4025513	3748758	93.12	1520609	40.56
29	NA19116_yale.bam	F	9693662	8872576	91.53	3716595	41.89
30	NA19119_yale.bam	M	3816178	3508443	91.94	1205991	34.37
31	NA19127_yale.bam	F	9353324	8563060	91.55	3313346	38.69
32	NA19128_yale.bam	M	9498521	8850191	93.17	3624291	40.95
33	NA19130_yale.bam	M	8914041	8200243	91.99	3550092	43.29
34	NA19131_yale.bam	F	8414765	7669283	91.14	3348908	43.67
35	NA19137_yale.bam	F	6574947	5798550	88.19	2658469	45.85
36	NA19138_yale.bam	M	9041280	8050926	89.05	3514539	43.65
37	NA19140_yale.bam	F	8970177	8098705	90.28	3490525	43.10
38	NA19141_yale.bam	M	9626823	8654012	89.89	3845564	44.44
39	NA19143_yale.bam	F	8381930	7619857	90.91	3164070	41.52
40	NA19144_yale.bam	M	8327861	7575682	90.97	3483974	45.99
41	NA19152_yale.bam	F	10247210	9327922	91.03	4141515	44.40
42	NA19153_yale.bam	M	8474542	7714514	91.03	3467783	44.95
43	NA19159_yale.bam	F	6382899	5684534	89.06	2559611	45.03
44	NA19160_yale.bam	M	9662020	8700495	90.05	3607606	41.46
45	NA19171_yale.bam	M	6154300	5529208	89.84	2389182	43.21
46	NA19172_yale.bam	F	7086910	6395504	90.24	2717398	42.49
47	NA19192_yale.bam	M	8552871	7637761	89.30	3324720	43.53
48	NA19193_yale.bam	F	9370200	8470026	90.39	3250051	38.37
49	NA19200_yale.bam	M	6213671	5642583	90.81	2437189	43.19
50	NA19201_yale.bam	F	9408711	8611509	91.53	3780209	43.90
51	NA19203_yale.bam	M	9630700	8685162	90.18	3461329	39.85
52	NA19204_yale.bam	F	6353169	5627854	88.58	2521069	44.80
53	NA19206_2_yale.bam	F	11010264	9624047	87.41	4179240	43.42
54	NA19207_yale.bam	M	11044860	9917916	89.80	4361614	43.98
55	NA19209_yale.bam	F	10301610	9403400	91.28	4086635	43.46
56	NA19210_yale.bam	M	9956027	9091087	91.31	3868188	42.55
57	NA19238_yale.bam	F	8406706	7657214	91.08	3267161	42.67
58	NA19239_yale.bam	M	9085407	8279008	91.12	3533765	42.68

Table S2. Gender-related genes in gene sets msY and XiE

msY	Gene	Description
1	RPS4Y1	ribosomal protein S4, Y-linked 1
2	ZFY	zinc finger protein, Y-linked
3	USP9Y	ubiquitin specific peptidase 9, Y-linked
4	DDX3Y	DEAD (Asp-Glu-Ala-Asp) box polypeptide 3, Y-linked
5	UTY	ubiquitously transcribed tetratricopeptide repeat containing, Y-linked
6	TMSB4Y	thymosin beta 4, Y-linked
7	NLGN4Y	neuroligin 4, Y-linked
8	TXLNG2P	taxilin gamma 2, pseudogene
9	EIF1AY	eukaryotic translation initiation factor 1A, Y-linked
10	RPS4Y2	ribosomal protein S4, Y-linked 2
11	PRKY	protein kinase, Y-linked, pseudogene
**XiE**	**Gene**	**Description**
1	EIF2S3	eukaryotic translation initiation factor 2, subunit 3 gamma, 52kDa
2	ZFX	zinc finger protein, X-linked
3	SH3BGRL	SH3 domain binding glutamic acid-rich protein like
4	PRKX	protein kinase, X-linked
5	NLGN4X	neuroligin 4, X-linked
6	STS	steroid sulfatase (microsomal), isozyme S
7	TBL1X	transducin (beta)-like 1X-linked
8	SLC25A6	solute carrier family 25 (mitochondrial carrier; adenine nucleotide translocator), member 6
9	ASMTL	acetylserotonin O-methyltransferase-like
10	DHRSX	dehydrogenase/reductase (SDR family) X-linked
11	CD99	CD99 molecule
12	ARSD	arylsulfatase D
13	RENBP	renin binding protein
14	FUNDC1	FUN14 domain containing 1
15	RPS4X	ribosomal protein S4, X-linked
16	GAB3	GRB2-associated binding protein 3
17	ARHGAP4	Rho GTPase activating protein 4

18	RAB9A	RAB9A, member RAS oncogene family
19	OFD1	oral-facial-digital syndrome 1
20	GPM6B	glycoprotein M6B
21	USP9X	ubiquitin specific peptidase 9, X-linked
22	DDX3X	DEAD (Asp-Glu-Ala-Asp) box polypeptide 3, X-linked
23	PIR	pirin (iron-binding nuclear protein)
24	CA5B	carbonic anhydrase VB, mitochondrial
25	L1CAM	L1 cell adhesion molecule
26	AP1S2	adaptor-related protein complex 1, sigma 2 subunit
27	CTPS2	CTP synthase 2
28	SYAP1	synapse associated protein 1
29	IKBKG	inhibitor of kappa light polypeptide gene enhancer in B-cells, kinase gamma
30	RBBP7	retinoblastoma binding protein 7
31	XIST	X inactive specific transcript (non-protein coding)
32	PLCXD1	phosphatidylinositol-specific phospholipase C, X domain containing 1
33	PPP2R3B	protein phosphatase 2, regulatory subunit B", beta
34	HDHD1	haloacid dehalogenase-like hydrolase domain containing 1
35	PNPLA4	patatin-like phospholipase domain containing 4
36	TCEANC	transcription elongation factor A (SII) N-terminal and central domain containing
37	TRAPPC2	trafficking protein particle complex 2
38	GEMIN8	gem (nuclear organelle) associated protein 8
39	CXorf38	chromosome X open reading frame 38
40	MED14	mediator complex subunit 14
41	UBA1	ubiquitin-like modifier activating enzyme 1
42	CDK16	cyclin-dependent kinase 16
43	KDM5C	lysine (K)-specific demethylase 5C
44	SMC1A	structural maintenance of chromosomes 1A
45	CHM	choroideremia (Rab escort protein 1)
46	EIF1AX	eukaryotic translation initiation factor 1A, X-linked
47	INE1	inactivation escape 1 (non-protein coding)
48	CD99P1	CD99 molecule pseudogene 1

Table S3. Type I error rates for different methods; α=0.05.

Test	msY	XiE	DES	Xi
edgeR_FM	≤0.001	≤0.001	≤0.001	0.215
DESeq_FM	≤0.001	≤0.001	≤0.001	0.231
eBayes_FM	≤0.001	≤0.001	≤0.001	0.266
edgeR_SM	≤0.001	≤0.001	≤0.001	0.579
DESeq_SM	≤0.001	≤0.001	≤0.001	0.448
eBayes_SM	≤0.001	≤0.001	≤0.001	0.424
edgeR_GM	≤0.001	≤0.001	≤0.001	0.004
DESeq_GM	≤0.001	≤0.001	≤0.001	0.003
eBayes_GM	≤0.001	≤0.001	≤0.001	0.006
N_RPKM	≤0.001	≤0.001	≤0.001	0.272
N_QQN	≤0.001	≤0.001	≤0.001	0.266
N_TMM	≤0.001	≤0.001	≤0.001	0.289
N_VOOM	≤0.001	≤0.001	≤0.001	0.264
WW_RPKM	≤0.001	≤0.001	≤0.001	0.754
WW_QQN	≤0.001	≤0.001	≤0.001	0.552
WW_TMM	≤0.001	≤0.001	≤0.001	0.745
WW_VOOM	≤0.001	≤0.001	≤0.001	0.764
KS_RPKM	≤0.001	≤0.001	≤0.001	0.792
KS_QQN	≤0.001	≤0.001	≤0.001	0.884
KS_TMM	≤0.001	≤0.001	≤0.001	0.587
KS_VOOM	≤0.001	≤0.001	≤0.001	0.856
ROAST	≤0.001	≤0.001	≤0.001	0.214
