# Supplementary material for: Comparative evaluation of gene set analysis approaches for RNA-Seq data
Source: BMC Bioinformatics. 2014 Dec 5;15(1):397. doi: 10.1186/s12859-014-0397-8 (PMC4265362; doi:10.1186/s12859-014-0397-8)
Supplement: Supplementary file 3 — Supplementary figures containing Supplementary figures S1-S13. [file 12859_2014_397_MOESM3_ESM.pdf]

Comparative evaluation of gene set analysis approaches for RNA-Seq data (Supplementary Figures)

Yasir Rahmatallah [1], Frank Emmert-Streib [2] and Galina Glazko [1]
[1] Division of Biomedical Informatics,
University of Arkansas for Medical Sciences,
Little Rock, AR 72205.
[2] Computational Biology and Machine Learning Laboratory,
Center for Cancer Research and Cell Biology,
School of Medicine, Dentistry and Biomedical Sciences,
Queen's University Belfast, 97 Lisburn Road, Belfast, BT9 7BL, UK.

October 30, 2014

List of Figures

S1 Density plots for the original Nigerian dataset and the Negative Binomial simulated counts before and after different normalizations. 2

S2 Histogram plots for the original Nigerian dataset and the Negative Binomial simulated counts before and after different normalizations. 3

S3 The power curves of multivariate tests with different normalizations when shift alternative hypothesis (H_1) holds true and the number of genes in pathways $p = 100$ ($N = 20$). 4

S4 The power curves of multivariate tests with different normalizations when shift alternative hypothesis (H_1) holds true and the number of genes in pathways $p = 16$ ($N = 40$). 5

S5 The power curves of multivariate tests with different normalizations when shift alternative hypothesis (H_1) holds true and the number of genes in pathways $p = 100$ ($N = 40$). 6

S6 The power curves of univariate tests with different P-values combining methods when shift alternative hypothesis (H_1) holds true and the number of genes in pathways $p = 100$ ($N = 20$). 7

S7 The power curves of univariate tests with different P-values combining methods when shift alternative hypothesis (H_1) holds true and the number of genes in pathways $p = 16$ ($N = 40$). 8

S8 The power curves of univariate tests with different P-values combining methods when shift alternative hypothesis (H_1) holds true and the number of genes in pathways $p = 100$ ($N = 40$). 9

S9 The power curves of different univariate tests with same P-values combining method when shift alternative hypothesis (H_1) holds true and the number of genes in pathways $p = 16$ ($N = 20$). 10

S10 The power curves of different univariate tests with same P-values combining method when shift alternative hypothesis (H_1) holds true and the number of genes in pathways $p = 100$ ($N = 20$). 11

S11 The power curves of different univariate tests with same P-values combining method when shift alternative hypothesis (H_1) holds true and the number of genes in pathways $p = 16$ ($N = 40$). 12

S12 The power curves of different univariate tests with same P-values combining method when shift alternative hypothesis (H_1) holds true and the number of genes in pathways $p = 100$ ($N = 40$). 13

S13 Venn diagrams showing the number of common pathways detected in the processed Nigerian dataset by multivariate tests with normalizations and gene-level GSA methods ($\alpha = 0.001$). (a) N-statistic with different normalizations and ROAST; (b) WW with different normalizations and ROAST; (c) KS with different normalizations and ROAST; (d) edgeR with different P-values combining methods; (e) DESeq with different P-values combining methods; (f) eBayes with different P-values combining methods; (g) univariate tests with FM; (h) univariate tests with SM; (i) univariate tests with GM. 14

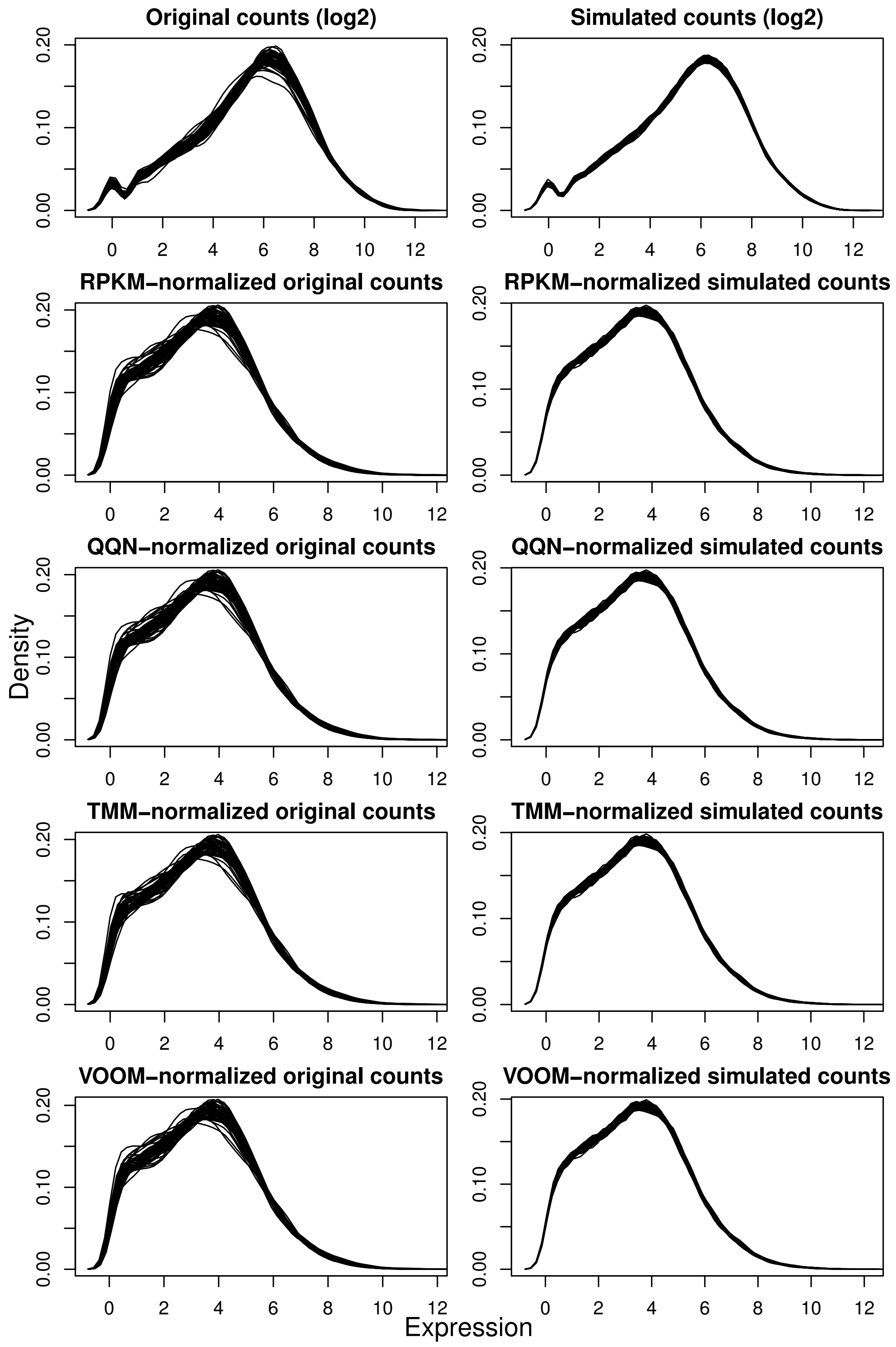

Figure S1: Density plots for the original Nigerian dataset and the Negative Binomial simulated counts before and after different normalizations.

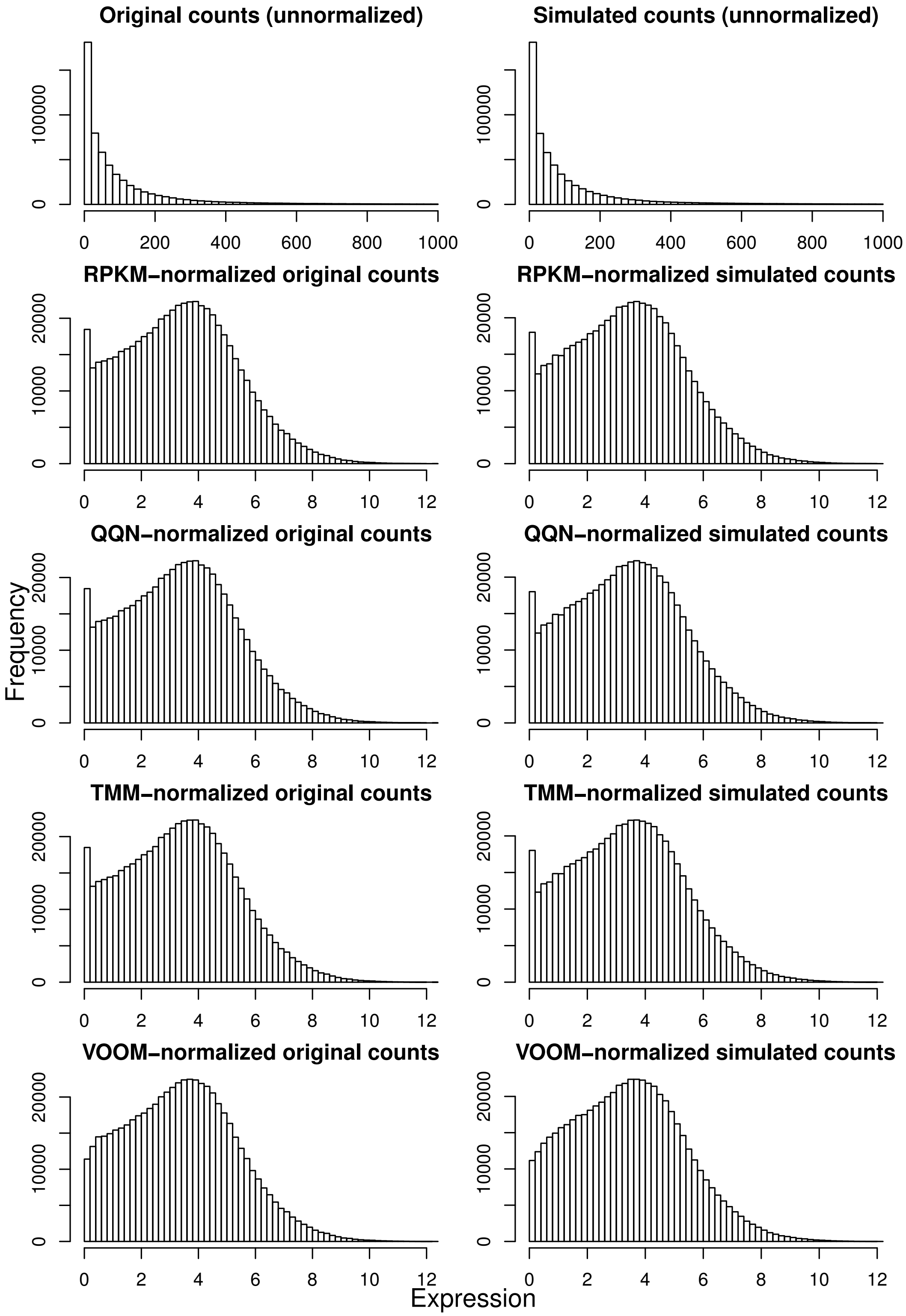

Figure S2: Histogram plots for the original Nigerian dataset and the Negative Binomial simulated counts before and after different normalizations.

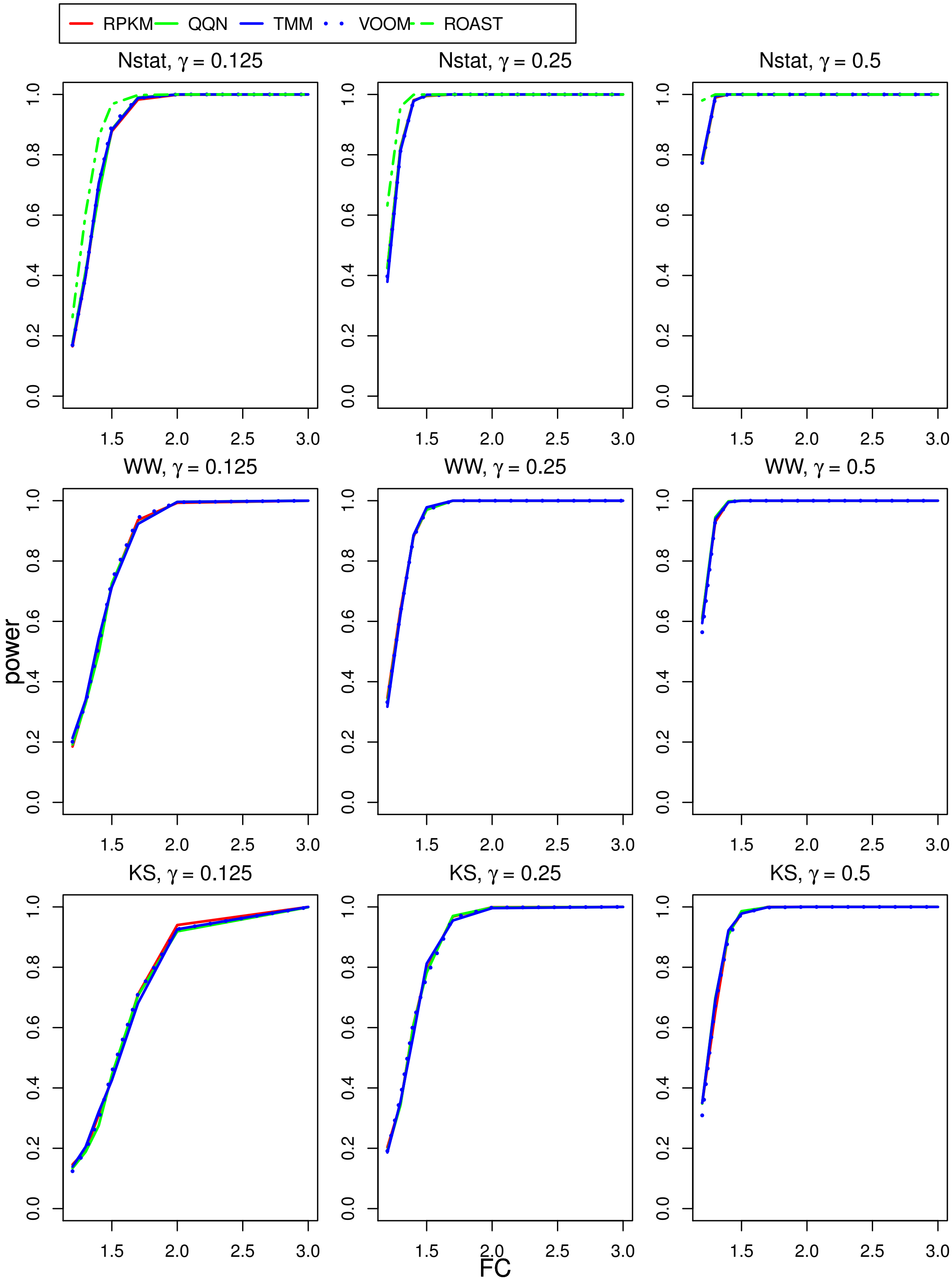

Figure S3: The power curves of multivariate tests with different normalizations when shift alternative hypothesis (H_1) holds true and the number of genes in pathways $p = 100$ ($N = 20$).

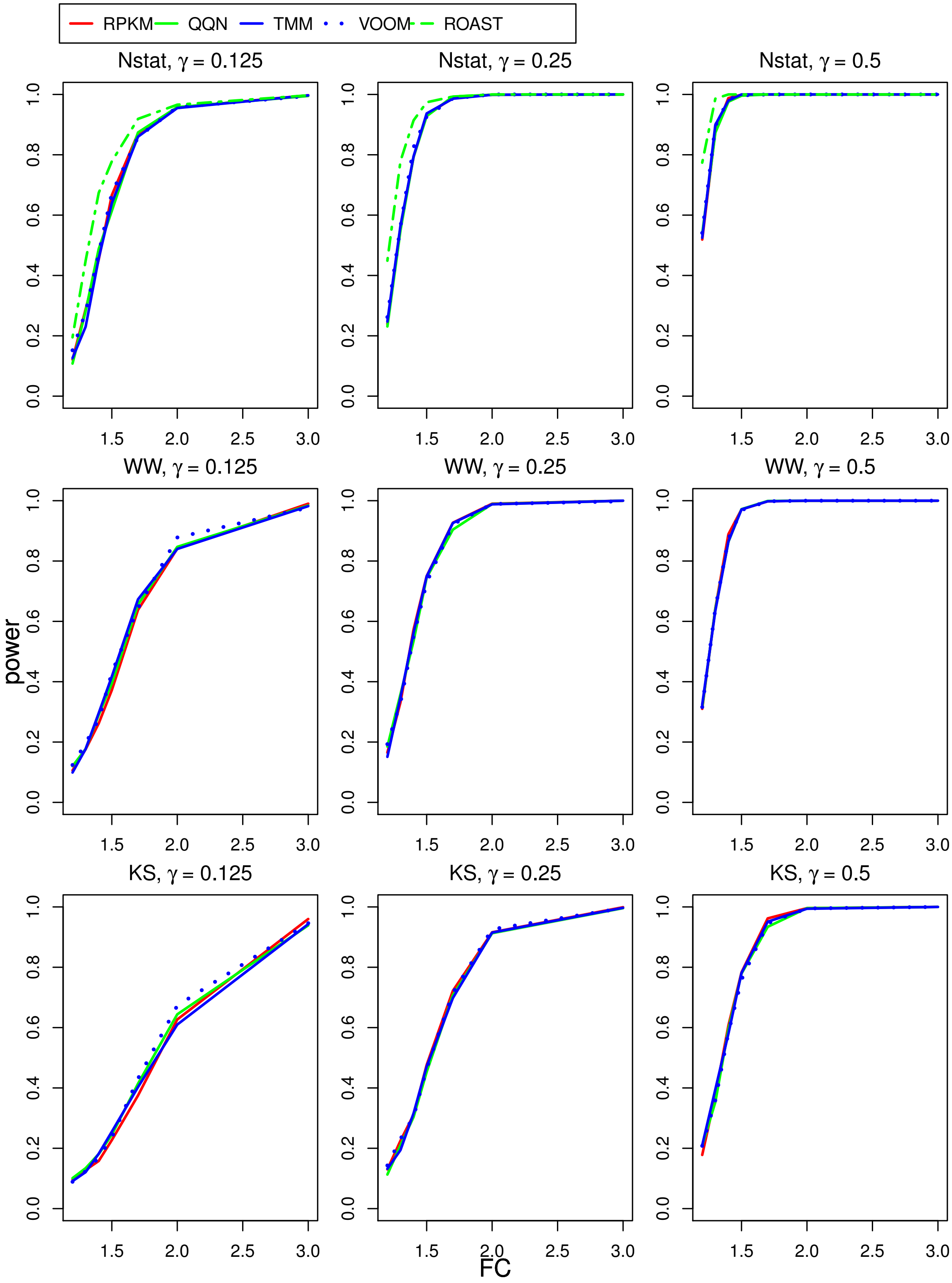

Figure S4: The power curves of multivariate tests with different normalizations when shift alternative hypothesis (H_1) holds true and the number of genes in pathways $p = 16$ ($N = 40$).

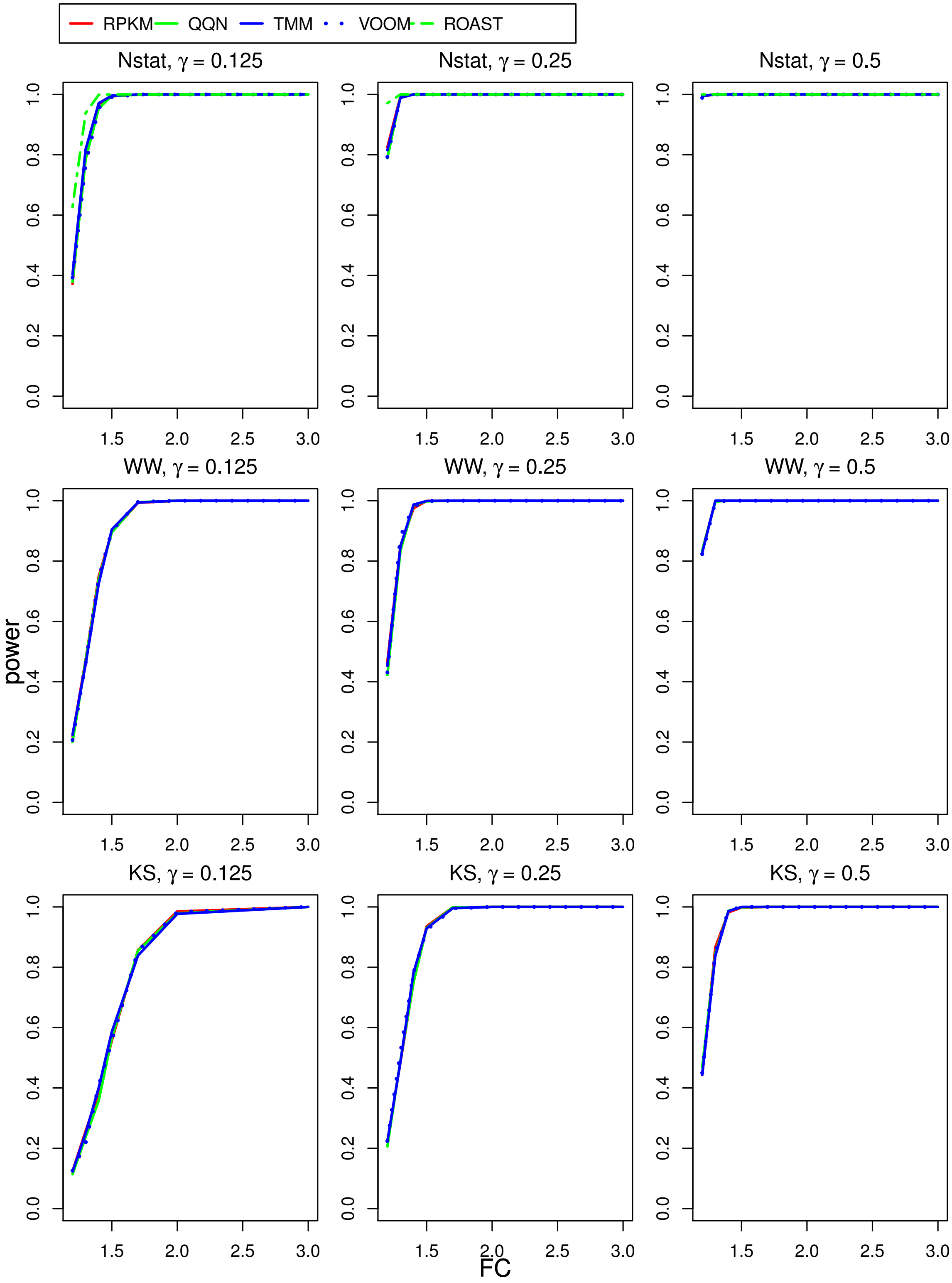

Figure S5: The power curves of multivariate tests with different normalizations when shift alternative hypothesis (H_1) holds true and the number of genes in pathways $p = 100$ ($N = 40$).

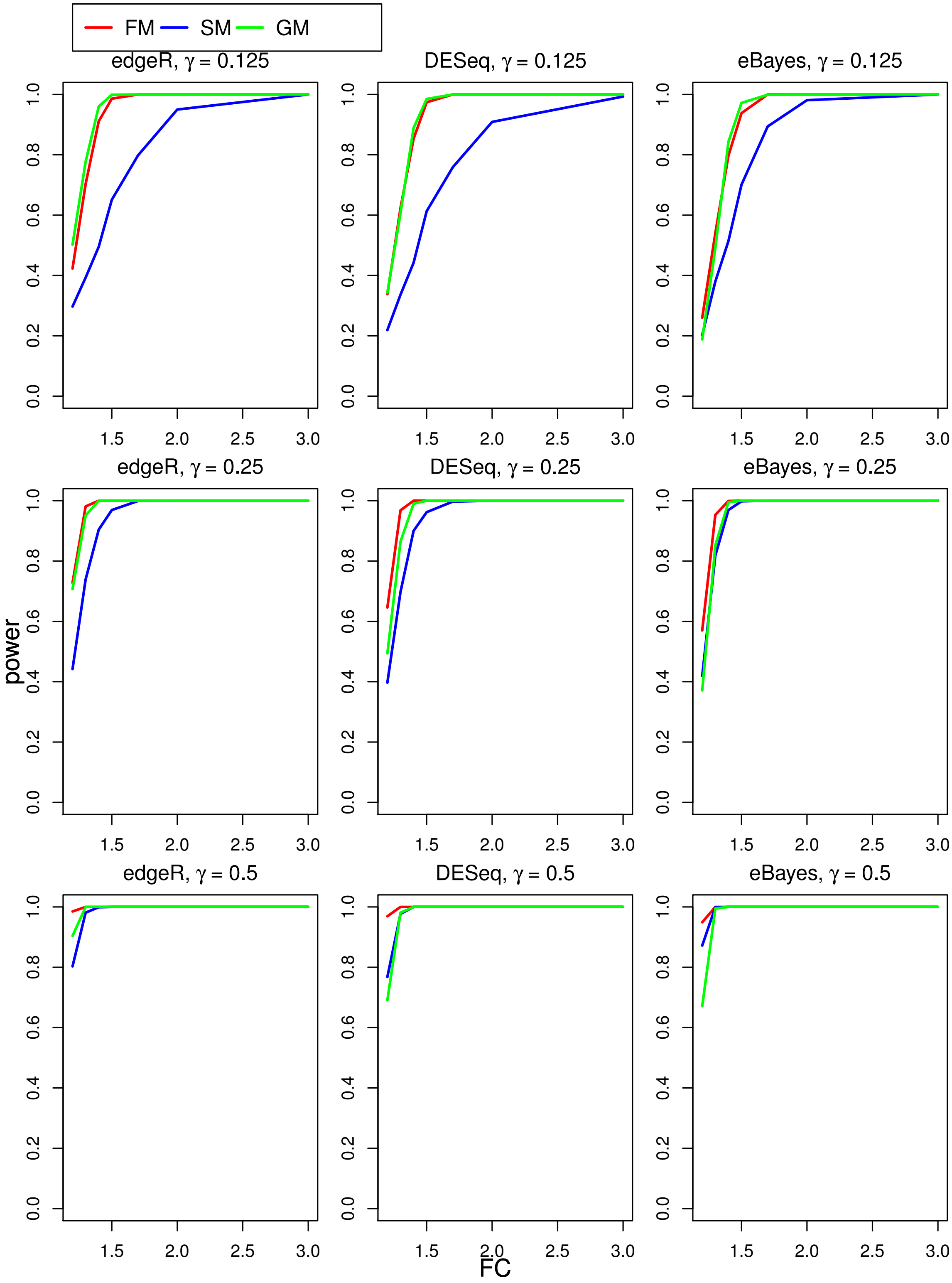

Figure S6: The power curves of univariate tests with different P-values combining methods when shift alternative hypothesis (H_1) holds true and the number of genes in pathways $p = 100$ ($N = 20$).

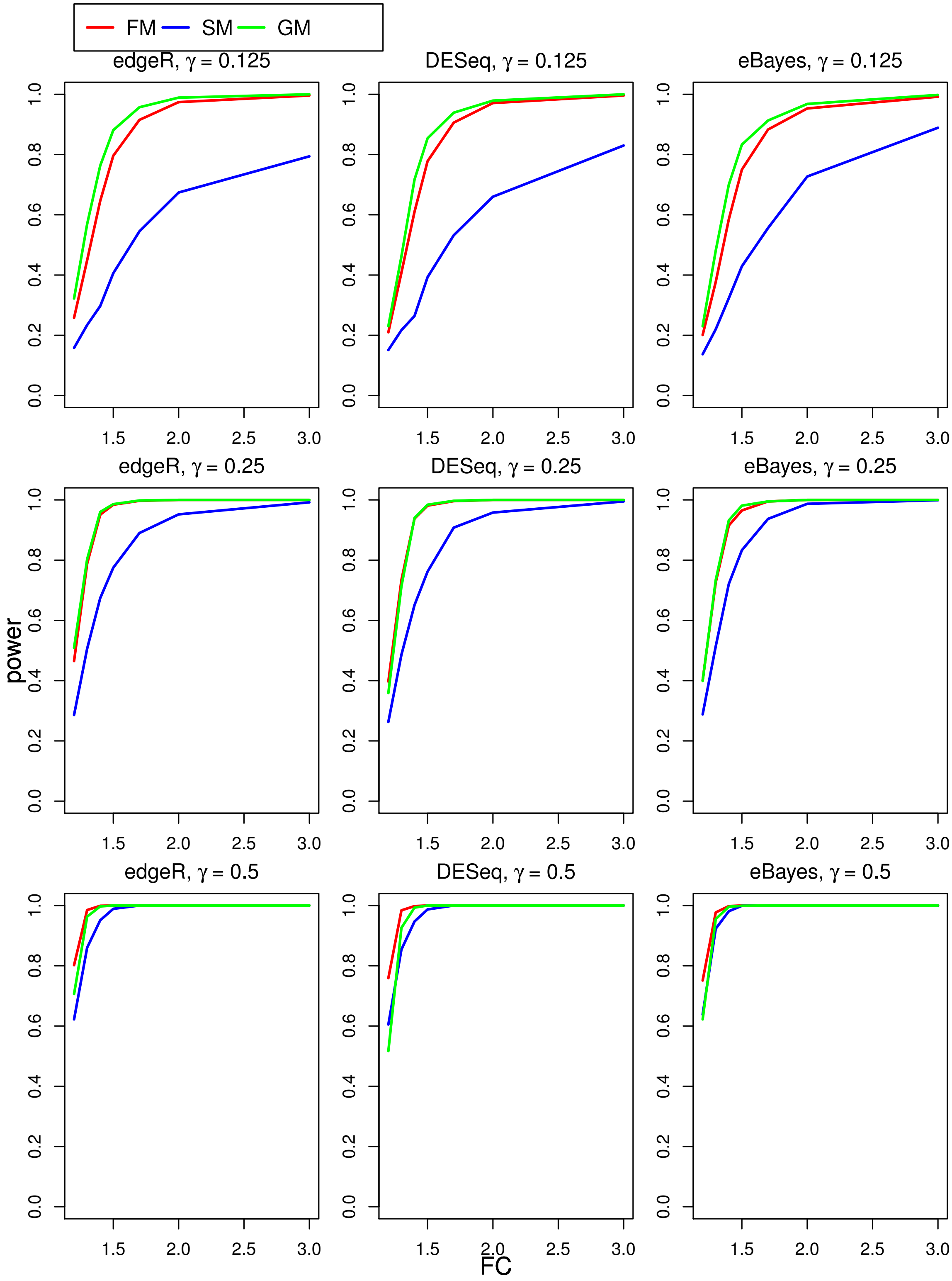

Figure S7: The power curves of univariate tests with different P-values combining methods when shift alternative hypothesis (H_1) holds true and the number of genes in pathways $p = 16$ ($N = 40$).

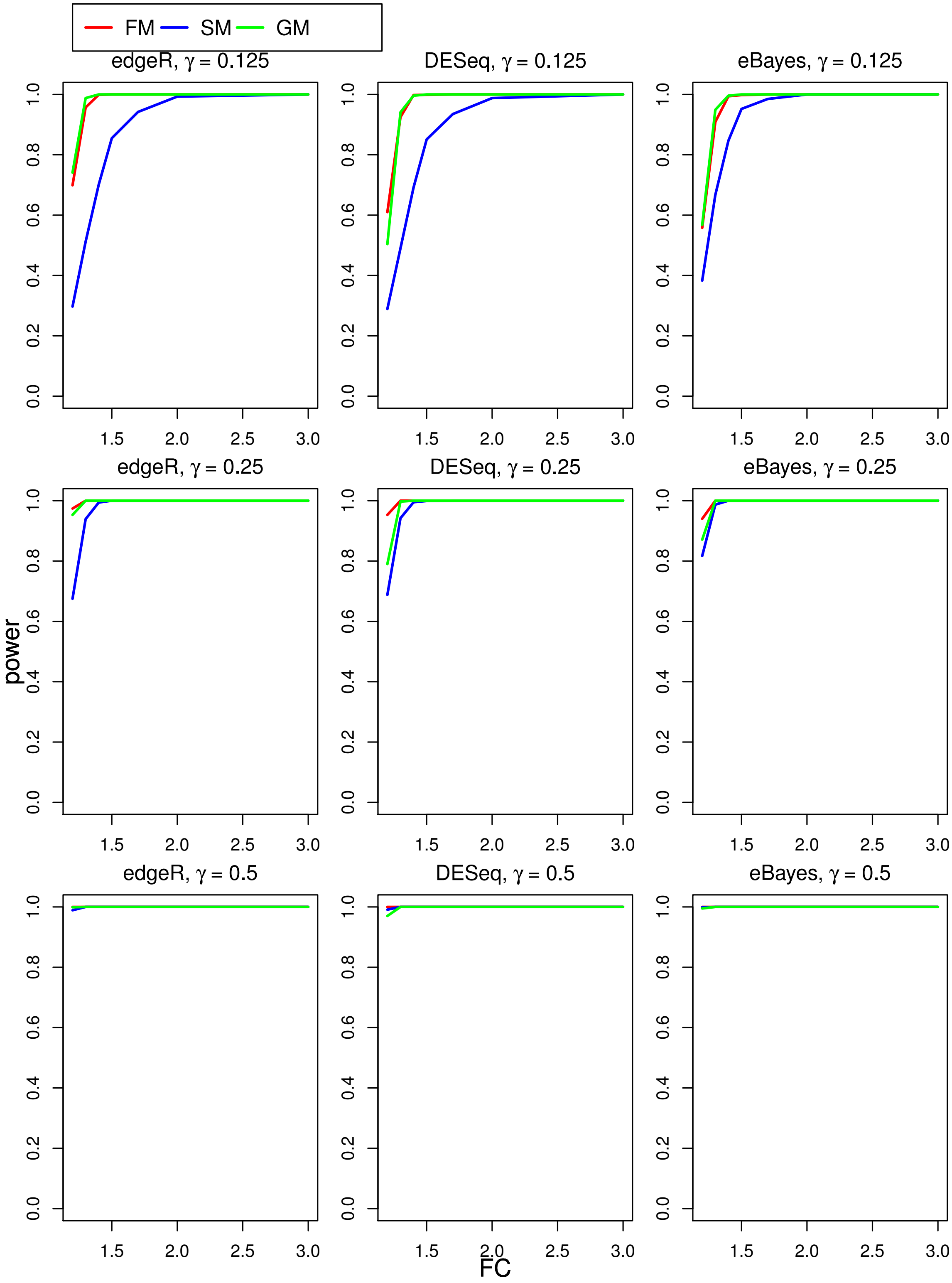

Figure S8: The power curves of univariate tests with different P-values combining methods when shift alternative hypothesis (H_1) holds true and the number of genes in pathways $p = 100$ ($N = 40$).

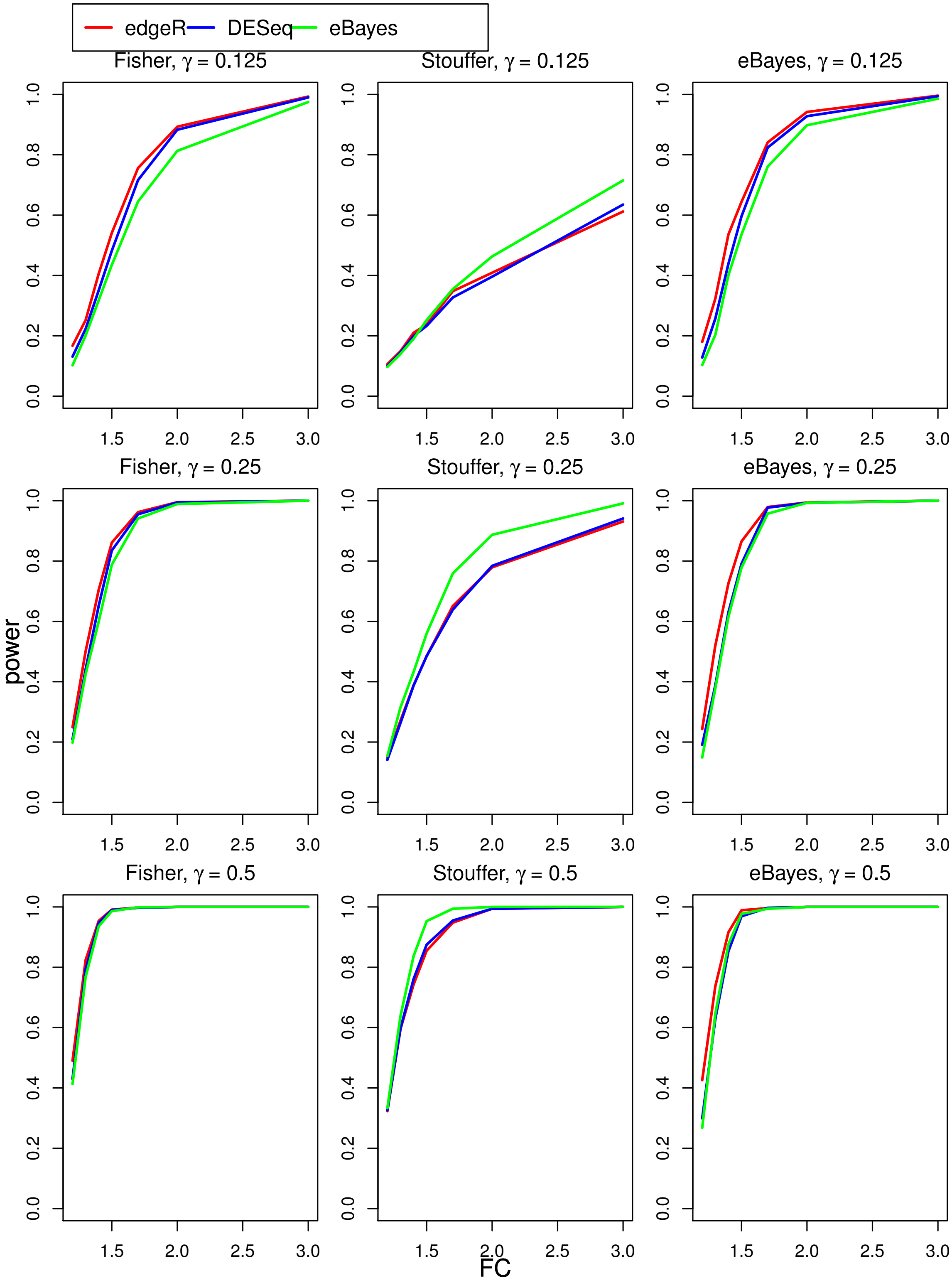

Figure S9: The power curves of different univariate tests with same P-values combining method when shift alternative hypothesis (H_1) holds true and the number of genes in pathways $p = 16$ $(N = 20)$.

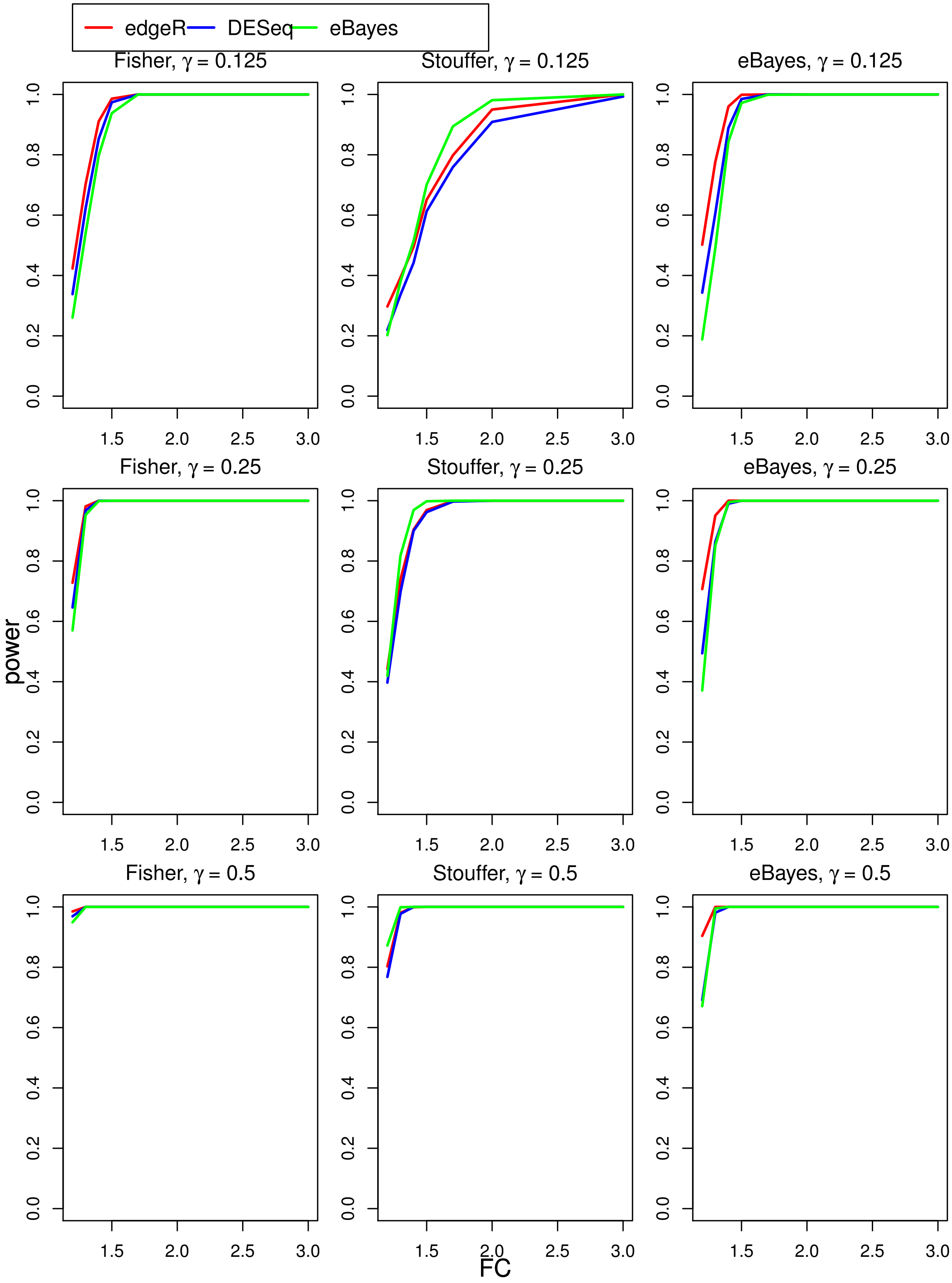

Figure S10: The power curves of different univariate tests with same P-values combining method when shift alternative hypothesis (H_1) holds true and the number of genes in pathways $p = 100$ ($N = 20$).

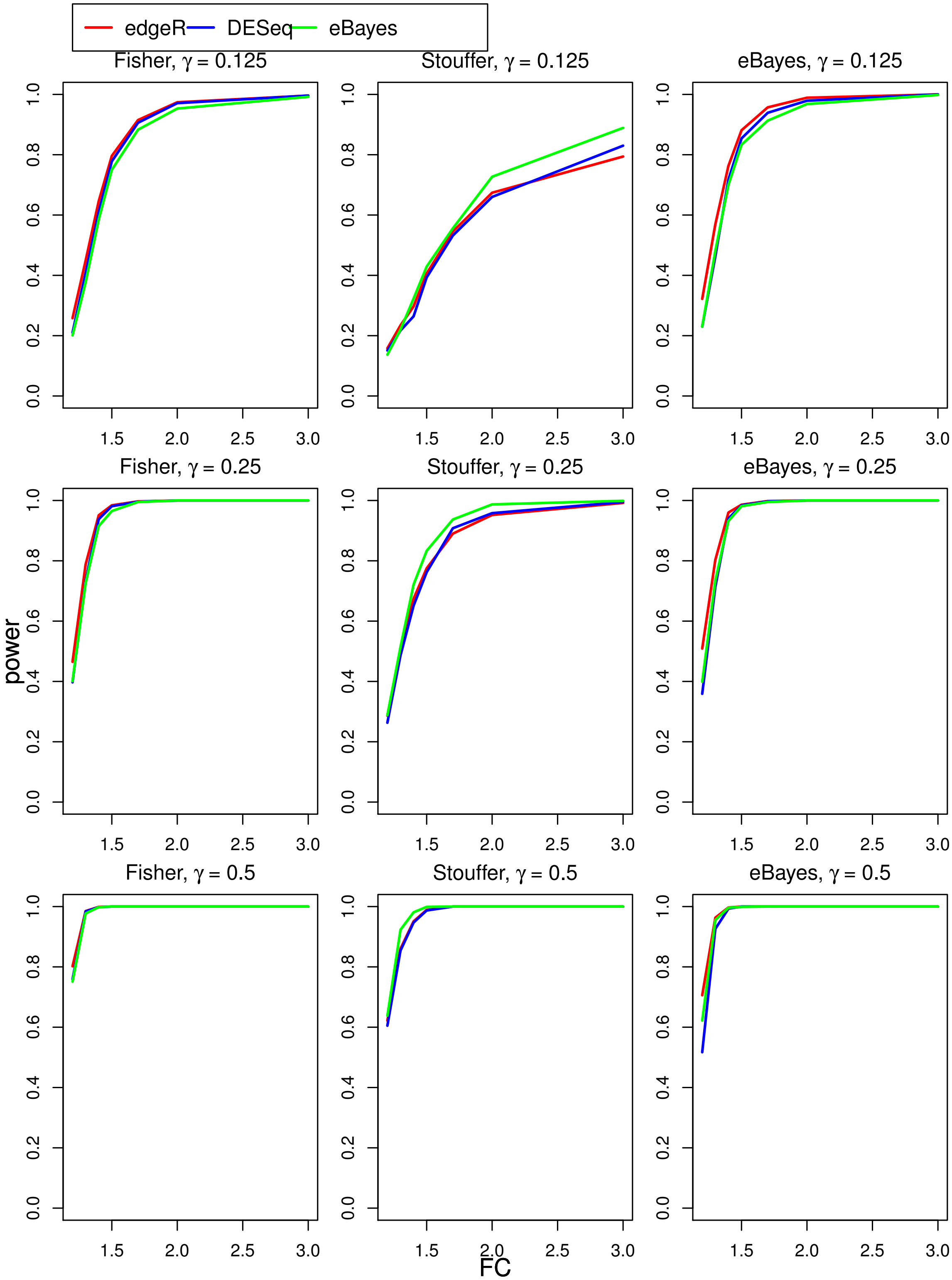

Figure S11: The power curves of different univariate tests with same P-values combining method when shift alternative hypothesis (H_1) holds true and the number of genes in pathways $p = 16$ $(N = 40)$.

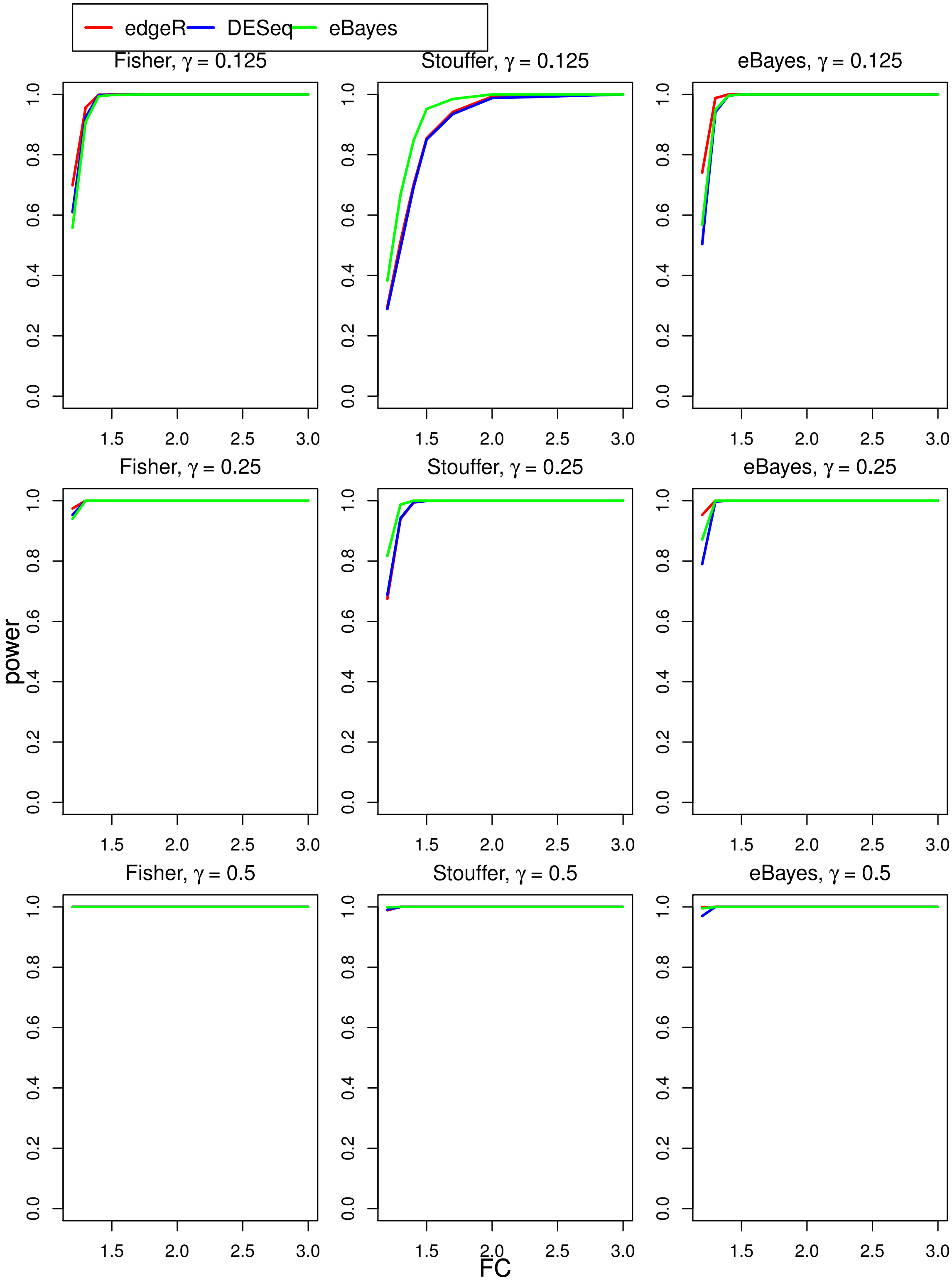

Figure S12: The power curves of different univariate tests with same P-values combining method when shift alternative hypothesis (H_1) holds true and the number of genes in pathways $p = 100$ ($N = 40$).

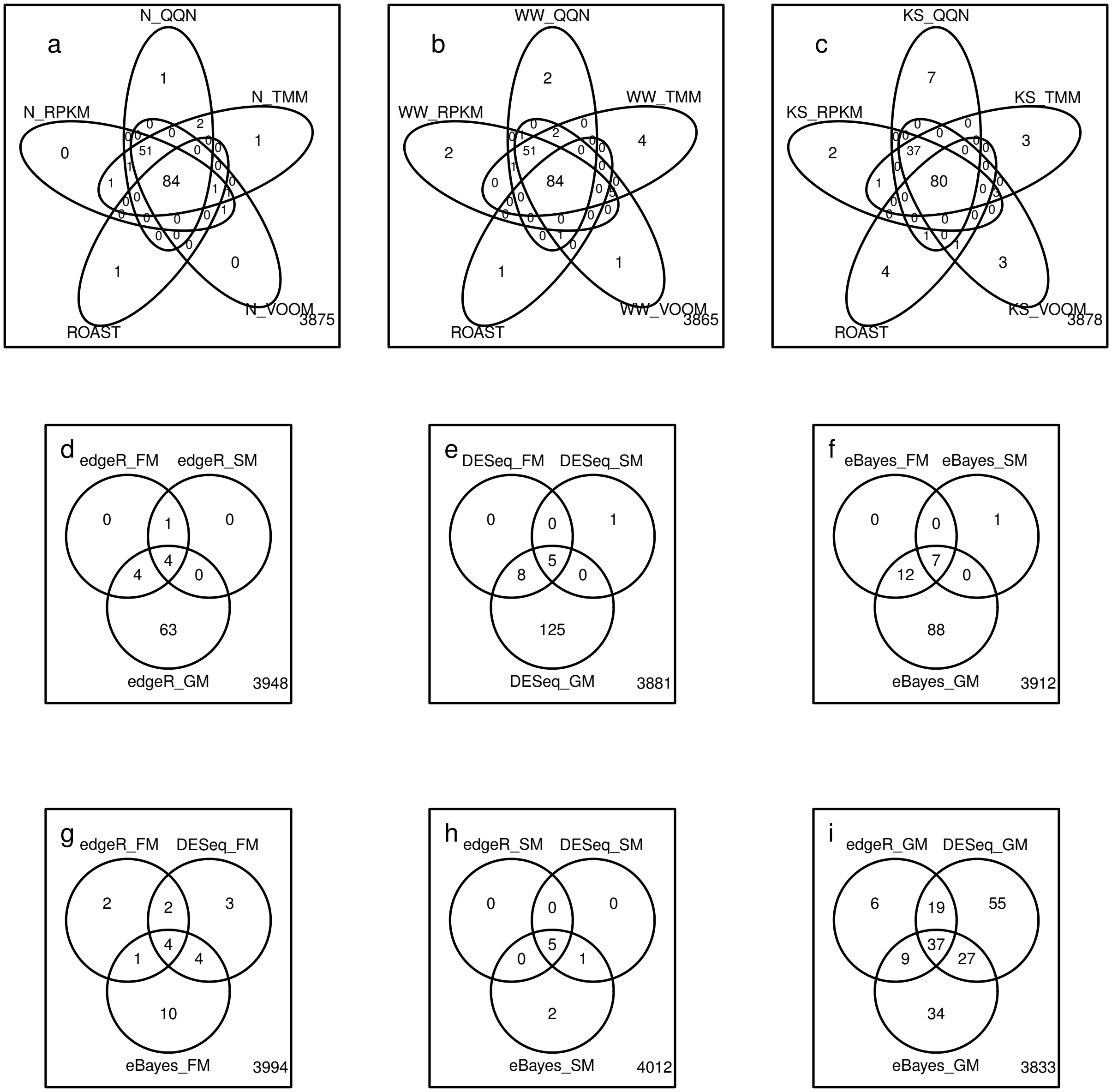

Figure S13: Venn diagrams showing the number of common pathways detected in the processed Nigerian dataset by multivariate tests with normalizations and gene-level GSA methods ($\alpha = 0.001$). (a) N-statistic with different normalizations and ROAST; (b) WW with different normalizations and ROAST; (c) KS with different normalizations and ROAST; (d) edgeR with different P-values combining methods; (e) DESeq with different P-values combining methods; (f) eBayes with different P-values combining methods; (g) univariate tests with FM; (h) univariate tests with SM; (i) univariate tests with GM.
